# Finite element analysis predicts Ca^2+^ microdomains within tubular-sarcoplasmic reticular junctions of amphibian skeletal muscle

**DOI:** 10.1038/s41598-021-93083-1

**Published:** 2021-07-13

**Authors:** Oliver J. Bardsley, Hugh R. Matthews, Christopher L.-H. Huang

**Affiliations:** 1 Physiological Laboratory, University of Cambridge, Downing Street, Cambridge, CB2 3EG UK; 2 Department of Biochemistry, University of Cambridge, Tennis Court Road, Cambridge, CB2 1QW UK

**Keywords:** Biophysics, Physiology

## Abstract

A finite element analysis modelled diffusional generation of steady-state Ca^2+^ microdomains within skeletal muscle transverse (T)-tubular-sarcoplasmic reticular (SR) junctions, sites of ryanodine receptor (RyR)-mediated SR Ca^2+^ release. It used established quantifications of sarcomere and T-SR anatomy (radial diameter $d=220 \, \mathrm{n}\mathrm{m}$; axial distance $w=12 \, \mathrm{n}\mathrm{m}$). Its boundary SR Ca^2+^ influx densities,${J}_{\mathrm{influx}}$, reflected step impositions of *influxes*, $\it {\Phi }_{\mathrm{influx}}={J}_{\mathrm{influx}}\left(\frac{\pi {d}^{2}}{4}\right),$ deduced from previously measured Ca^2+^ signals following muscle fibre depolarization. *Predicted* steady-state T-SR junctional edge [Ca^2+^], [Ca^2+^]_edge,_ matched reported corresponding *experimental* cytosolic [Ca^2+^] elevations given diffusional boundary *efflux* $\it \it {\Phi }_{\mathrm{efflux}}=\frac{D [ {{{\mathrm{Ca}}^{2+}}}]_{\mathrm{edge}}}{\lambda } (\pi dw),$ established cytosolic Ca^2+^ diffusion coefficients $(D = 4 \times {10}^{7} \mathrm{nm}^{2}/\mathrm{s})$ and exit length $\lambda = 9.2 \, \mathrm{n}\mathrm{m}$. Dependences of *predicted* [Ca^2+^]_edge_ upon ${J}_{\mathrm{influx}}$ then matched those of *experimental* [Ca^2+^] upon Ca^2+^ release through their entire test voltage range. The resulting model consistently predicted elevated steady-state T-SR junctional ~ µM-[Ca^2+^] elevations radially declining from maxima at the T-SR junction centre along the entire axial T-SR distance. These [Ca^2+^] heterogeneities persisted through 10^4^- and fivefold, variations in *D* and *w* around, and fivefold reductions in *d* below, control values, and through reported resting muscle cytosolic [Ca^2+^] values, whilst preserving the flux conservation ($\it \it {\Phi }_{\mathrm{influx}}={\Phi }_{\mathrm{efflux}})$ condition, ${\left[\mathrm{C}{\mathrm{a}}^{2+}\right]}_{\mathrm{edge}}=\frac{\lambda {dJ}_{\mathrm{influx}}}{4Dw}$. Skeletal muscle thus potentially forms physiologically significant ~ µM-[Ca^2+^] T-SR microdomains that could regulate cytosolic and membrane signalling molecules including calmodulin and RyR, These findings directly fulfil recent experimental predictions invoking such Ca^2+^ microdomains in observed regulatory effects upon Na^+^ channel function, in a mechanism potentially occurring in similar restricted intracellular spaces in other cell types.

## Introduction

Intracellular endoplasmic or sarcoplasmic reticular (SR) membrane systems gating store Ca^2+^ release into the cytosol following surface membrane activation, often involving ryanodine receptor (RyR) activation, occur widely amongst cell types. These include both excitable (skeletal, cardiac and smooth muscle, and cerebellar Purkinje^1–3^, hippocampal^4^ and other central nervous system neurones^5,6^) and non-excitable, including thrombocyte, cell types^7,8^. These intracellular membranes often form appositions with surface membrane with proximities (< 10–30 nm) permitting direct protein–protein/lipid interaction^9,10^ though not accommodating entire organelles. Their intervening electron-dense cytosol could also reflect local concentrations of proteins, lipids or ions. Electron microscopic sections can reveal parallel alignments extending over ~ 100–400 nm distances without fusion of the component membranes potentially offering restricted diffusion spaces permitting ion, including Ca^2+^, accumulation and microdomain formation.

In skeletal and cardiac muscle, following surface membrane propagation, Na^+^ channel mediated action potentials are conducted into the cellular interior at regular intervals along the muscle length through electrically continuous transverse (T-) tubular membranes. At specific regions, these come geometrically close (~ 12 nm) to, whilst remaining electrically isolated from, terminal cisternal membranes of the SR Ca^2+^ store. The resulting T-SR triad and dyad junctions are strategic to excitation–contraction coupling^11–13^. In cardiac muscle, tubular membrane Ca^2+^ influx through voltage-activated dihydropyridine receptors (DHPR2; Cav﻿1.2) activates SR Ca^2+^ release by cardiac-type SR RyR2^14^. In skeletal muscle, Ca_v﻿_1.1 DHPR1 conformational changes themselves allosterically activate directly coupled RyR1^15^.

Released Ca^2+^ then diffuses into the cytosol activating nearby myofilaments and consequent muscle contraction, prior to its SR Ca^2+^-ATPase mediated re-sequestration at longitudinal as opposed to T-SR membrane sites. However, recent evidence implicates Ca^2+^ in further regulatory functions involving T and SR membrane proteins. Skeletal and cardiac muscle Na^+^ channels (Na_v_1.4 and Na_v_1.5) possess potential Ca^2+^ and calmodulin (CaM) binding modulatory sites^16^. Here Ca^2+^ can bind directly to the Na_v_ C-terminal domain at one or more of its EF-like hand motifs^17^, or indirectly, through Ca^2+^/CaM binding, to its isoleucine-glutamine (IQ) domain region following initial Ca^2+^ binding to EF-hand motifs on CaM^18,19^ or to a site between Na_v_ domains III and IV^20^. Voltage-gated Na^+^ channels additionally contain sites phosphorylatable by Ca^2+^/CaM-regulated CaM kinase II (CaMKII)^21^, and by protein kinase C^22^. In vitro single-cell patch clamp studies reported that elevating [Ca^2+^]_i_ to ~ 2 µM by rapid Ca^2+^ photo-release or overspill from neighbouring Ca^2+^ channels, reduced Na^+^ current, *I*_Na_, in Na_v_1.4-transfected HEK293 cells and skeletal muscle cell lines. These effects were abrogated by intracellular BAPTA^23^, or mutations involving either the CaM Ca^2+^-binding-EF hands or the Nav﻿1.4 C-terminal IQ domain^19,23,24^.

Intact native murine skeletal muscle fibres were also studied by a loose patch clamping technique that minimized perturbations in intracellular Ca^2+^ signalling^25^. Activation or inhibition of SR Ca^2+^ release then respectively reduced or increased Na^+^ current, *I*_Na_. The latter were accomplished by direct pharmacological RyR1 activation by caffeine or 8‐(4‐chlorophenylthio)‐2′‐O‐methyladenosine‐3′,5′‐cyclic monophosphate (8-CPT), or RyR1 inhibition by dantrolene. Dantrolene pretreatment further abrogated the downregulatory effects of caffeine or 8-CPT on *I*_Na_^26,27^. Murine cardiac muscle showed similar effects when SR Ca^2+^ release was enhanced either by 8-CPT challenge, or with the RyR2-P2328S genetic modification^28,29^. In contrast, cyclopiazonic acid (CPA) increased skeletal muscle *I*_Na_, while preserving its time courses, steady-state half-maximum voltages and steepness factors. CPA pre-treatment also abrogated the effects of caffeine^30^. Yet previous Ca^2+^ fluorescence studies performed in rat soleus and oesophageal striated muscle using fluo-3-AM and fura-PE3-AM had reported that both caffeine and CPA increased bulk cytosolic [Ca^2+^]^31–34^. However, in contrast to modifying RyR-mediated SR Ca^2+^ release, CPA inhibits SR Ca^2+^-ATPase (SERCA)-mediated cytosolic Ca^2+^ re-uptake^31,32^. The consequent store Ca^2+^ depletion would then be expected to reduce, rather than increase, RyR1-mediated Ca^2+^ influx into the T-SR junction^30^.

These paradoxical findings prompted suggestions that RyR1-mediated Ca^2+^ release took place into a microdomain in the vicinity of both the SR RyR1 and the T-tubular membrane Na_v_1.4 and that the local elevation in Ca^2+^ concentration, [Ca^2+^]_TSR_, would then modify Na_v﻿_1.4 function^30^. Such a hypothesis would predict contrasting increases and decreases in local microdomain [Ca^2+^] following caffeine, and dantrolene or CPA challenge. The narrow, ~ 12 nm T-SR junctions that could form spaces with restricted intracellular diffusion close to the RyR1 Ca^2+^-release sites might be implicated in such microdomain formation. These could result in changes in local in vivo [Ca^2+^]_TSR_, distinct from those of [Ca^2+^]_i_ in the remaining bulk cytosol. This could explain the contrasting actions of RyR1 agonists and RyR1 or SERCA antagonists on *I*_Na_ through correspondingly contrasting effects on local Ca^2+^ or Ca^2+^/CaM levels, to which the Na_v_1.4 would be directly or indirectly exposed, despite their similar effects on bulk cytosolic [Ca^2+^]_i_^33,34^.

Direct experimental explorations for such [Ca^2+^]_TSR_ microdomains possibly using fluorescent Ca^2+^ indicator methods need to address the small dimensions and dispersed nature of the T-SR compartment^35–37^. The present complementary approach applies diffusional modelling techniques^38^ to explore the physical parameters permitting accumulation or depletion of released SR Ca^2+^ within the T-SR junction. It demonstrated that established anatomical and physiological features related to skeletal muscle excitation–contraction coupling are physically compatible with generation of significant Ca^2+^ microdomains in both activated and resting muscle fibres. We then discuss their possible physiological effects both in myocyte T-SR junctions and in similar membrane appositions in other cell types.

## Results

### T-SR junction structure represented using a formal geometric model

We employed anatomical, optical and electron microscope quantifications of sarcomere and T-SR junction structure from amphibian twitch fibres as the muscle type for which the fullest data are available^39–44^. This provided the required details of T-tubular-sarcoplasmic reticular (T-SR) junction anatomy for the modelling studies (Table 1). First, the reported values of sarcomere length *l*, fibre diameter *a*, and relative tubular (T) to surface (S) membrane area reflected in the ratio of their respective capacitances *C*_T_/*C*_S_, yielded the sarcomeric surface membrane area:

1 $$A_{{\text{S}}} = \pi al$$

and the total tubular membrane surface area:

2 $$\it \it {A}_{\mathrm{T}}=\left(\frac{{C}_{T}}{{C}_{S}}\right)\mathrm{\pi }al$$

**Table 1:** Structural characteristics of amphibian skeletal muscle fibres and transverse tubular-sarcoplasmic reticular (T-SR) junctions.

Name of variable	Definition	Value (physiological units)	Value (SI units)				
**Geometrical characteristics: muscle fibre**							
Length of sarcomere^41^	$l$	3.65 μm	3.65 × 10^–6^ m				
Diameter of fibre^39^	*a*	100 μm	1.00 × 10^–4^ m				
Surface membrane capacitance^39^	*C*_S_	1.0 μF/cm^2^	1.00 × 10^–2^ F/m^2^				
T-Tubular membrane capacitance^40^	*C*_T_	5.0 μF/cm^2^	5.00 × 10^–2^ F/m^2^				
**Derived variables used in modelling**							
Ratio of T-tubular to surface membrane capacitance	(*C*_T_*/C*_**S**_)	5.0	5.0				
Sarcomere surface membrane area	*A*_S_ = *πal*	1147 μm^2^	1.147 × 10^–9^ m^2^				
Sarcomere tubular membrane area	*A*_T_ = (*C*_T_*/C*_S_)π*al*	5733 μm^2^	5.733 × 10^–9^ m^2^				
Sarcomere volume	*V* = π*a*^*2*^*l*/*4*	2.87 × 10^4^ μm^3^	2.87 × 10^–14^ m^3^				
**Geometrical characteristics: T-SR junction**							
Proportion of T-tubular membrane area apposed to triad junctions^44^	*ξ*	0.3	0.3				
Width of T-SR Junction^44^	*w*	12 nm	1.2 × 10^–8^ m				
Diameter of sarcoplasmic reticular (SR) terminal cisternae^42,43^	*d*	220 nm	2.20 × 10^–7^ m				
**Derived variables used in modelling**							
SR membrane area of T-SR junction	π*d*^*2*^/*4*	38,013.27 nm^2^	3.8013 × 10^–14^ m^2^				
Area at edge of T-SR junction	π*dw*	8293.804 nm^2^	8.2938 × 10^–14^ m^2^				
Ratio of volume of T-SR spaces to that of whole cell	*ξwA*_T_*/V*	7.20 × 10^–4^	7.20 × 10^–4^				
Tubular membrane area abutted by T-SR junction	*A*_TSR_ = *ξA*_T_	1720 μm^2^	1.720 × 10^–9^ m^2^				
Total number of T-SR junctions in one sarcomere	*4A*_TSR_*/(*π*d*^2^*)*	4.5248 × 10^4^	4.5248 × 10^4^				
Total number of T-SR junctions in unit volume of muscle	*N*_TSR_ = 16*ξC*_T_/*(*π*d*^2^*a C*_**S**_)	1.5784 × 10^15^/dm^3^	1.5784 × 10^18^/m^3^				

Flux properties, previous experimental results^45^							
Test membrane potential, *E* (mV)		− 45	− 40	− 30	− 20	−10	0
Maximum rate of [Ca^2+^] increase, d[Ca^2+^]/d*t* (μmol/(dm^3^s))		3.55	18.0	90.0	120.0	170.0	180.0
Peak cytosolic calcium concentration, [Ca^2+^]_max_ (μmol/dm^3^)		0.135	0.511	1.711	2.427	2.858	3.161
**Computed boundary conditions over range of varied test voltages: T-SR junction**							
Ca^2+^ flux density into T-SR junction, *J*_influx_ (mol/(nm^2^s))		5.92 × 10^–26^	3.00 × 10^–25^	1.50 × 10^–24^	2.00 × 10^–24^	2.83 × 10^–24^	3.00 × 10^–24^
Ca^2+^ flux into T-SR junction, *Φ*_influx_ (mol/s)		2.25 × 10^–21^	1.14 × 10^–20^	5.70 × 10^–20^	7.60 × 10^–20^	1.07 × 10^–19^	1.14 × 10^–19^
Ca^2+^ diffusion coefficient in vivo^46,47^, *D* (nm^2^/s)		4 × 10^7^	4 × 10^7^	4 × 10^7^	4 × 10^7^	4 × 10^7^	4 × 10^7^
Exit length, *λ* (nm)		9.2	9.2	9.2	9.2	9.2	9.2

**Figure 1:**
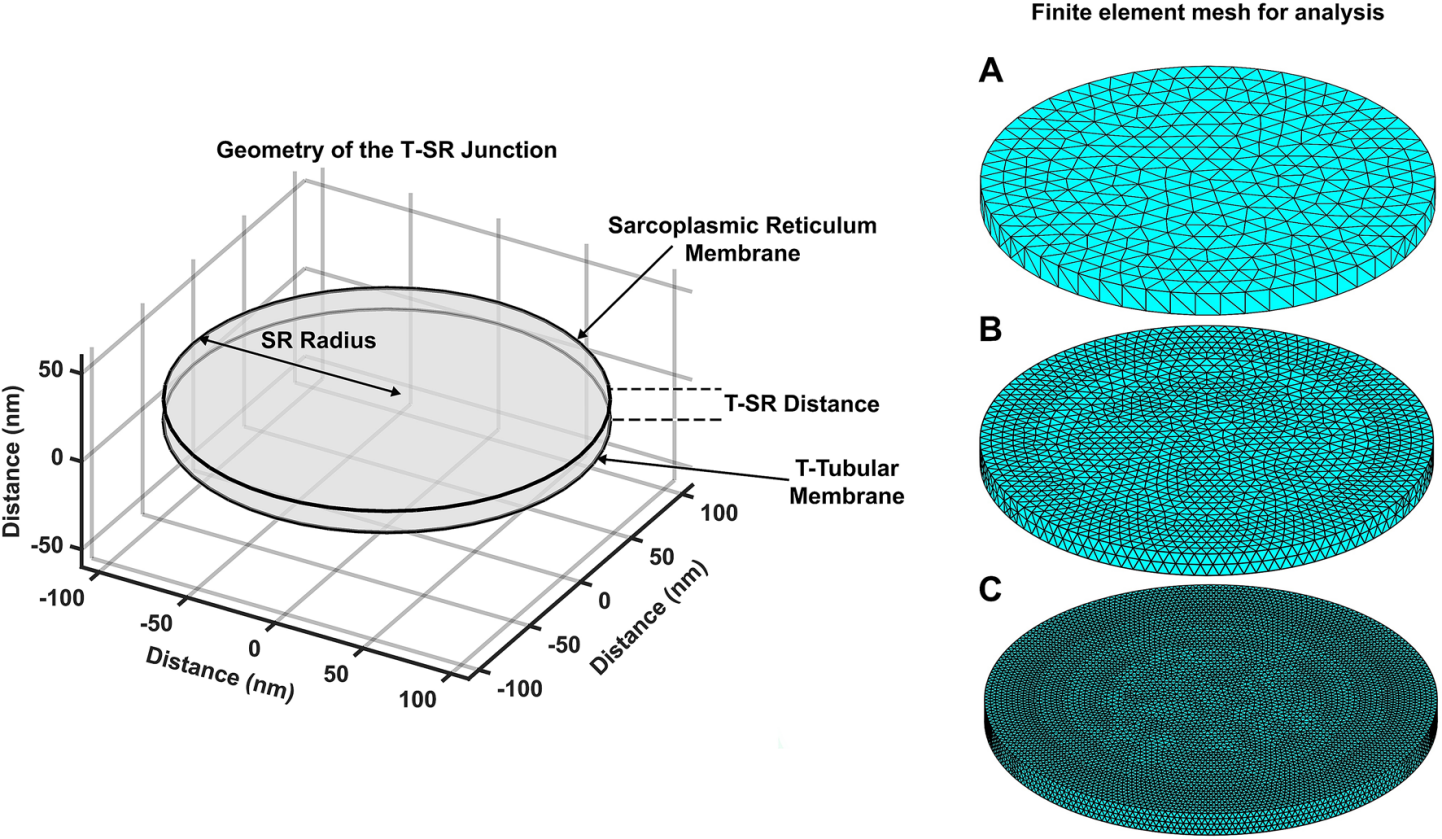
T-SR junction structure represented using a formal geometric model divided into finite elements. Left: Geometrical representation of formalized T-SR junction represented as two disks, respectively of tubular (T) and sarcoplasmic reticular (SR) membranes, of diameter, *d* = 220 nm, within the radial (*xy*) plane, separated along an axial (*z*) direction by a T-SR distance, *w* = 12 nm. Right: Superimposition of finite elements dividing T-SR junction geometry into tetrahedral ‘elements’ with specified maximum lengths of (**A**) 12 nm, or 100%, (**B**) 6 nm or 50%, and (**C**) 3 nm or 25%, of the T-SR distance respectively. Reduced maximum tetrahedral lengths increase the number of elements, within which each time-dependent solution is generated, increasing spatial resolution in the solution concentration profile.

Morphometric electron micrographic estimates of the proportion *ξ* of the total tubular membrane area *A*_T_ accounted for by T-SR junctions^44^ then gave the total T-tubular membrane area contributing to T-SR junction structures *A*_TSR_ = *ξA*_T_*.*,*w*,*d* of SR membrane enclosed in a single T-SR junction. The latter was accordingly modelled as a circularly symmetrical disk-shaped space of dimensions *w* = 12 nm and *d* = 220 nm (Fig. 1,

### Ca^2+^ diffusion into and through a single T-SR junction modelled by finite element analysis

The finite element analysis of steady state Ca^2+^ diffusion through a single T-SR space used the circular ends of the geometry defined above to represent its respective T-tubular and SR membranes. The rim separating their edges connected the T-SR and remaining intracellular spaces. *Influx* boundary conditions were supplied by a steady-state and uniform Ca^2+^ influx density *J*_influx_ across the SR membrane face of each individual T-SR junction:

3 $${\Phi }_{\mathrm{influx}}={J}_{\mathrm{influx}}\left(\frac{\pi {d}^{2}}{4}\right)$$

In contrast, the T-tubular face represented a zero-flux boundary surface. The Ca^2+^ then diffuses through and leaves the T-SR space at the rim with diffusion coefficient *D*:

4 $$\frac{\partial \left[\mathrm Ca^{2+}\right]}{\partial t}-\nabla \cdot \left({\mathrm {D}}\nabla \left[{\mathrm {Ca}}^{2+}\right]\right)+{\mathrm{ p}}\left[{\mathrm {Ca}}^{2+}\right]=0$$

Away from the boundaries where *p* = 0:

5 $$\frac{\partial \left[Ca^{2+}\right]}{\partial t}=\nabla \cdot \left(D\nabla [Ca^{2+}]\right)$$

At the *efflux* boundary, the constant *p* is proportional to the diffusion coefficient *D*, *p* = *D*/*λ*, giving:

6 $$\frac{\partial \left[{Ca^{2+}}\right]}{\partial t}=\nabla \cdot \left(D \nabla [Ca^{2+}]\right)-\left(\frac{D}{\lambda }\right)\left[{Ca^{2+}}\right]=0$$

This edge region thus incorporates a first-order efflux Neumann BC describing diffusional Ca^2+^ efflux *Φ*_efflux_ leaving the T-SR space across its edge area π*dw*, giving efflux:

7 $$\it {\Phi }_{\mathrm{efflux}}={J}_{efflux}(\mathrm{\pi }dw)$$

Its efflux density *J*_efflux_ is proportional to the edge Ca^2+^ concentration [Ca^2+^]^TSR^_edge_:

8 $${J}_{\mathrm{efflux}}=\frac{D{\left[Ca^{2+}\right]}_{\mathrm{edge}}}{\lambda }$$

This gives efflux equation:

9 $$\it {\Phi }_{\mathrm{efflux}}=\frac{D{\left[Ca^{2+}\right]}_{\mathrm{edge}}}{\lambda }({\pi }dw)$$

Its Fick’s Law constant *D*/*λ* comprises the Ca^2+^ diffusion coefficient *D* and empirical exit length *λ*. The latter provides a geometrical parametrization of the re-uptake of the dissipated Ca^2+^ from a well stirred cytosolic compartment by SERCA activity without saturation. The *λ* term represents the only free parameter in the entire modelling analysis.

Diffusional processes within the T-SR junction were represented by superimposing a finite element mesh upon the T-SR junction geometry using PDE Toolbox, dividing that 3D geometry into fine tetrahedral elements of specified maximum length *χ* (Fig. 1, Right panels A–C). Different mesh sizes progressively divided the T-SR space into (A) 12 nm, (B) 6 nm and (C) 3 nm tetrahedral elements; finer mesh sizes were used where modelling investigated axial in addition to radial [Ca^2+^] gradients and in duplicate runs matching different mesh sizes to validate those used in the reconstructions. The FEM and MATLAB solved the equations specified for the system and specified input parameters producing a [Ca^2+^] dataset in the form of an array, whose spatial resolution was determined by the fineness of the mesh, set by the maximum element length *χ*. Its steady state solutions satisfied the overall conservation condition,

10 $$\it {\Phi }_{\mathrm{i}\mathrm{n}\mathrm{f}\mathrm{l}\mathrm{u}\mathrm{x}}={\Phi }_{\mathrm{e}\mathrm{f}\mathrm{f}\mathrm{l}\mathrm{u}\mathrm{x}}$$

between influx and efflux boundaries, yielding the condition (see Eqns. 3 and 9):

11 $${J}_{\mathrm{i}\mathrm{n}\mathrm{f}\mathrm{l}\mathrm{u}\mathrm{x}}\left(\frac{\pi {d}^{2}}{4}\right)=\frac{D{\left[\mathrm{Ca}^{2+}\right]}_{\mathrm{e}\mathrm{d}\mathrm{g}\mathrm{e}}}{\lambda }(\mathrm{\pi }dw)$$

This condition was used in checks for the steady state condition in detailed explorations of the effect of specific parameters that follow.

### Sarcoplasmic reticulum Ca^2+^ release producing Ca^2+^ microdomains characterized by radial concentration gradients in the T-SR junction

The modelling process first sought three-dimensional (3D) reconstructions of radial steady state [Ca^2+^] distributions resulting from diffusional processes using the computational T-SR parameters listed in Table 1. Ca^2+^ release influx densities into each individual T-SR junction, *J*_influx,_ used in the MATLAB program PDE Toolbox, could be related to previously reported^45^ experimental initial rates of SR Ca^2+^ release d[Ca^2+^]/d*t*. The latter would give a Ca^2+^ flux into the sarcomere cytosolic volume *V* of $\left\{\frac{\mathrm{d}\left[{\mathrm{C}\mathrm{a}}^{2+}\right]}{\mathrm{d}t}\right\}V$ in turn corresponding to a flux density into each T-SR junction of:

12 $${J}_{\mathrm{i}\mathrm{n}\mathrm{f}\mathrm{l}\mathrm{u}\mathrm{x}}=\frac{\left\{\frac{\mathrm{d}\left[{Ca}^{2+}\right]}{\mathrm{d}t}\right\}V}{\xi {A}_{T}}$$

Antipyrylazo III absorbances in amphibian skeletal muscle fibres subject to voltage clamp steps from a − 90 mV resting to a 0 mV test membrane potential^45^ reported a value of d[Ca^2+^]/d*t* = 180 μmol/(dm^3^ s). The Ca^2+^ then diffuses through the T-SR space down its resulting concentration gradients with the diffusion constant *D* = 4.0 × 10^7^ nm^2^/s previously reported for amphibian skeletal muscle^46–48^. The Ca^2+^ finally leaves the T-SR junction space effluxing into surrounding cytosol at the edge of the T-SR junction across diffusional area π*dw* at a rate driven by [Ca^2+^]^TSR^_edge_. This proved close and proportional to reported experimental peak cytosolic Ca^2+^ concentration, [Ca^2+^]_max_ at the explored 0 mV test voltage with the use of an exit length value *λ* = 9.2 nm^45^.

An overall rate constant describing the dependence of the summed Ca^2+^ fluxes upon [Ca^2+^]^TSR^_edge_ could be determined using the predicted number of T-SR junctions in unit muscle volume,

13 $${N}_{\mathrm{T}\mathrm{S}\mathrm{R}}=\frac{16\xi \left(\frac{{C}_{\mathrm{T}}}{{C}_{\mathrm{S}}}\right)}{{\pi ad}^{2}}$$

The total Ca^2+^ efflux into unit muscle volume is then:

14 $$\it {N}_{TSR}{\Phi }_{efflux}=\frac{16\xi w\left(\frac{{C}_{T}}{{C}_{S}}\right)D{\left[Ca^{2+}\right]}_{edge}}{\lambda ad}$$

The constant of proportionality describing this linear dependence on [Ca^2+^]^TSR^_edge_ is then.

$$\frac{16\xi Dw\left(\frac{{C}_{\mathrm{T}}}{{\mathrm{C}}_{\mathrm{S}}}\right)}{\lambda ad}=5.69 \, {\mathrm{s}}^{-1}$$

This resulting rate constant is smaller than but comparable to experimental rate constants describing eventual SR resequestration of the released cytosolic Ca^2+^. Thus, previous experimental studies suggested rate constants for such unsaturable SR Ca^2+-^ATPase mediated Ca^2+^ uptake around 22.3 ± 8.14/s under similarly steady state conditions where Ca^2+^ binding to remaining, saturable, fast-exchanging cytosolic binding sites was constant^49^.

**Figure 2:**
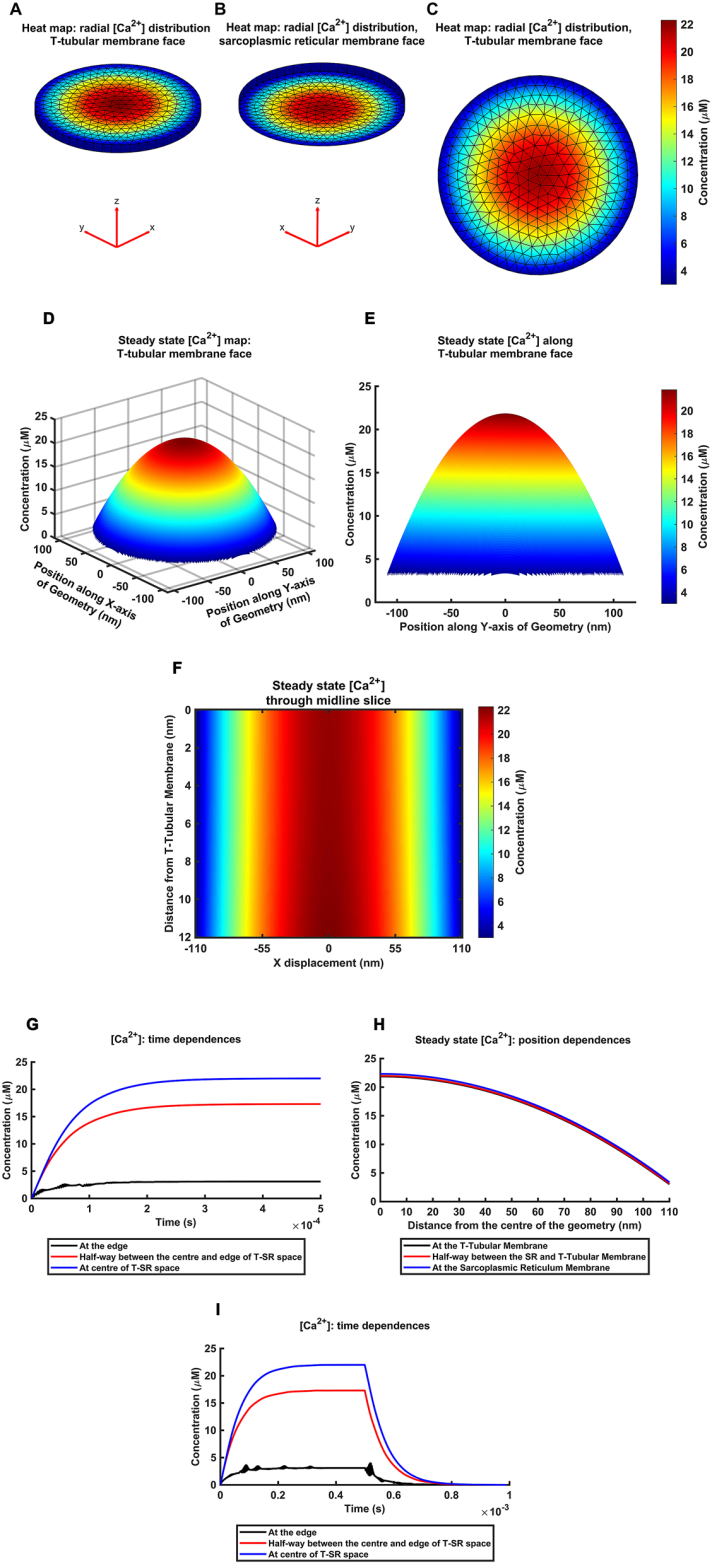
Sarcoplasmic reticulum Ca^2+^ release produces Ca^2+^ microdomains in the T-SR model. (A-C) Heat map with overlaid 12 nm finite element mesh demonstrating steady state radial [Ca^2+^] gradients following application of Ca^2+^ influx density, *J*_influx_, expected from a depolarizing step from the resting to a 0 mV test voltage in voltage clamped amphibian muscle fibre as described in previous reports^45^. Highest [Ca^2+^] is at the T-SR centres with ~ fivefold concentration reductions between the centre and edge in both (**A**), (**C**) T-tubular membrane and (**B**) SR membrane face. Edge concentrations are close between the two membranes. (**D**–**F**) The Ca^2+^ microdomains extend the entire axial T-SR distance. This is demonstrated in heat maps of: (**D**) radial [Ca^2+^] (vertical, *z*-axis) over the T-tubular membrane surface (*xy*-plane), more closely quantified by (**E**) midline axial section map of [Ca^2+^] taken along the *y*-axis. [Ca^2+^] plotted against radial distance along the T-tubular membrane in both *x* and *y* axes in (**D**) and in the *y* axis in (**E**). The jagged base of the surface plot is the result of interpolation of results from a cylindrical geometry. (**F**) [Ca^2+^] heatmap through midline axial section through the T-SR junction. Vertical axis plots axial (*z*) distance from the T-tubular membrane within the T-SR junction. Horizontal (*x*) axis plots radial distance from the T-SR junction centre. Colours represent the resulting steady state [Ca^2+^]. Note uniformity in concentration along the *z* axis and fivefold gradients along the *x* axis. Colour bars in (**D**) and (**E**), and (**F**), show [Ca^2+^] as represented in the heat maps. (**G**–**I**) Time course of microdomain [Ca^2+^] solutions at selected positions within the T-SR junction. (**G**) Solutions for [Ca^2+^] at the edge (black), centre (blue) of the T-SR junction and midway between these two (red lines) plotted against time following initiation of the Ca^2+^ influx, *J*_influx_ from the SR face of the T-SR junction. Initial instabilities in plots observed in the edge traces reflect stiff-stable properties of the solutions. (**H**) Variations in steady state [Ca^2+^] with radial distance from the centre of the T-SR junction parallel to the radial axis at the T-tubular membrane, the SR membrane, or halfway between these. (**I**) [Ca^2+^] plotted with time following the onset of Ca^2+^ influx, but with its termination after 0.5 ms.

Figure 2A–C map the predicted [Ca^2+^] through the radial geometry of the T-SR junction. The false colour maps reconstruct perspective steady-state [Ca^2+^] at the (A) tubular and (B) SR membrane faces, and (C) en face [Ca^2+^] at the tubular membrane face of the T-SR junction. Between elements where a solution has not been calculated, the result is interpolated automatically according to the coarseness of the mesh. These maps show a significant [Ca^2+^] heterogeneity or microdomain across the radial axis resulting in a > fivefold difference between centre and edge T-SR junction [Ca^2+^]. These resulting Ca^2+^ microdomains extended the entire T-SR distance mapping [Ca^2+^] gradients onto the tubular membrane surface. The colour map (Fig. 2D) shows a surface plot, with radial *x* and *y* axes representing the width and length of the T-tubular membrane abutted by SR and the axial *z* axis the [Ca^2+^] at that point, displaying [Ca^2+^] nonuniformities extending across the T-tubular membrane.

Figure 2E demonstrates an axially sliced Ca^2+^ microdomain extending to the edge of the T-SR junction with [Ca^2+^] radially falling ~ fivefold over ~ 100 nm from the T-SR junction centre. Figure 2F maps steady state [Ca^2+^] within a section taken along a midline axial plane through the T-SR junction. It illustrates diffusional effects following SR Ca^2+^ release along the bottom of the frame and the resulting nearly axially uniform [Ca^2+^] and radial [Ca^2+^] gradients running towards the T-SR junction edge.

### Steady state microdomain [Ca^2+^] quantified with position within the T-SR junction

The Ca^2+^ microdomain represented above was quantified from the array of [Ca^2+^] values at each element with time following imposition of the Ca^2+^ influx density *J*_influx_; missing data between element solutions were interpolated from nearby nodal solutions. To quantify [Ca^2+^] with time in the microdomain, each finite element in the mesh was assigned a number referenced using a command retrieving the solution from the node closest to a specified 3-dimensional Cartesian co-ordinate (https://www.mathworks.com/help/pde/ug/heat-transfer-problem-with-temperature-dependent-properties.html). The solution from the node closest to selected points representing the edge and centre of the geometry, and half-way between these was plotted against time through the time trajectory of the computation (Fig. 2G). These typically reached steady state over a ~ 0.2 ms exponential timecourse following the onset of the imposed *J*_influx,_ a timescale ~ 1–2 orders of magnitude shorter than experimentally measured Ca^2+^ transients in vivo^45^. The resulting steady state [Ca^2+^] at axial distances within the T-SR junction could additionally be obtained by interpolation for plotting against radial distance from the centre of the T-SR junction (Fig. 2H). Comparing [Ca^2+^] timecourses with imposition and termination of the Ca^2+^ fluxes confirmed that [Ca^2+^] recovered back to its initial starting value, as expected for a first-order diffusional system (Fig. 2I). Finally, solutions obtained using varying 6 nm and 3 nm mesh sizes gave [Ca^2+^] microdomain characteristics in close agreement, validating the computational parameters used in our finite element analysis (Supplementary Table S1).

### Persistent Ca^2+^ microdomains with [Ca^2+^] graded with Ca^2+^ flux densities through varied test voltages

The T-SR junction model was then tested against varying *J*_influx_ holding constant the remaining diffusional, *D* and *λ*, and T-SR geometrical parameters, *d* and *w* (Supplementary Table S2). The *J*_influx_ values used were calculated from previous experimental d[Ca^2+^]/d*t* produced by varying steps to physiologically activating depolarizing test voltages (Table 1). Microdomain T-SR centre and [Ca^2+^]_edge_ changes were then compared with corresponding experimental peak changes in intracellular Ca^2+^ concentrations [Ca^2+^]_max_, at the same test voltage^45^.

**Figure 3:**
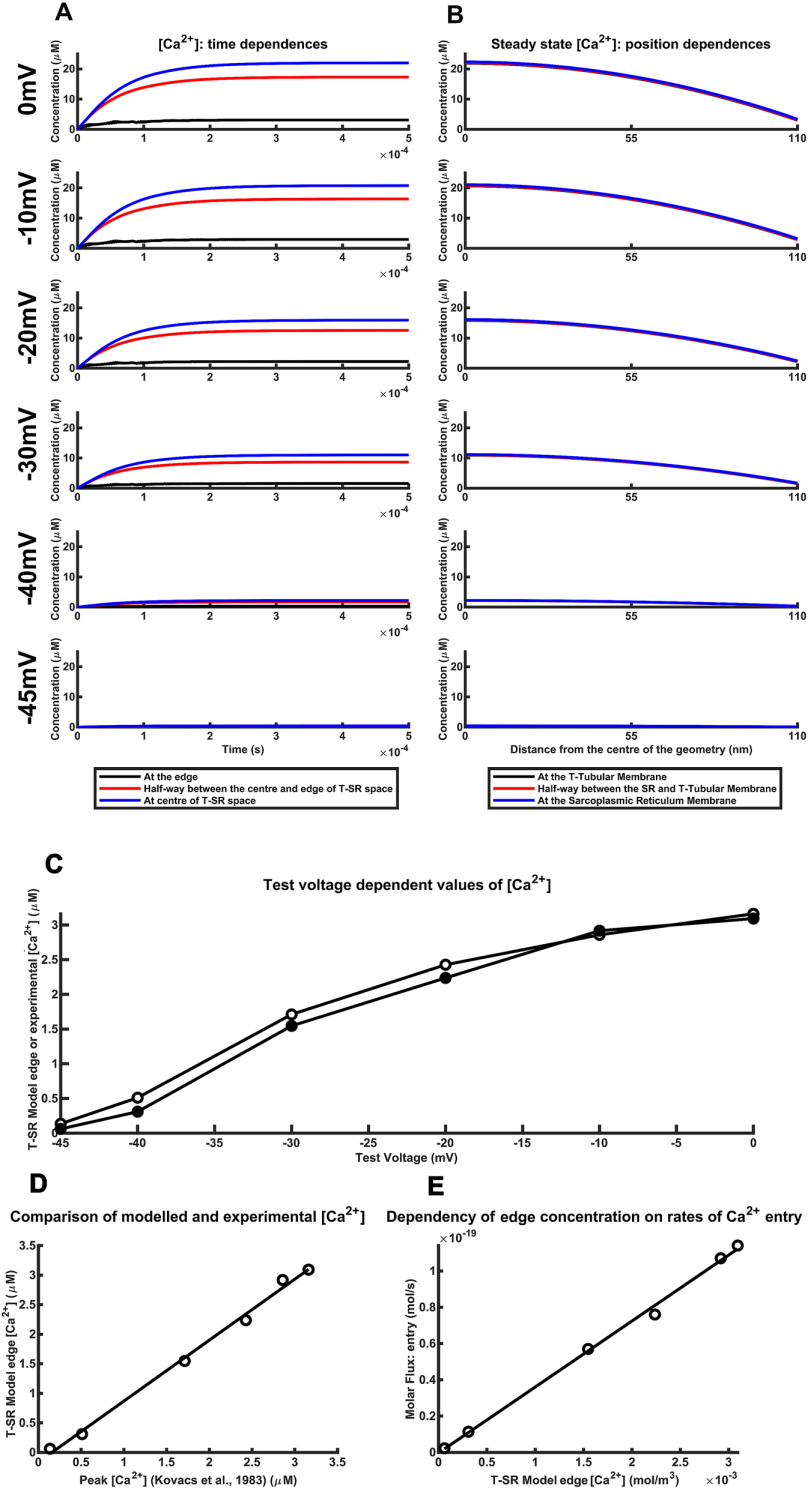
Ca^2+^ microdomains reconstructed using varied Ca^2+^ flux densities derived from experimental Ca^2+^ transients in response to test voltage steps. (**A**, **B**) Persistent Ca^2+^ microdomains with [Ca^2+^] magnitudes graded with experimentally derived Ca^2+^ influx densities *J*_influx_ corresponding to varying experimental test voltage steps applied to amphibian muscle fibres^45^. (**A**) [Ca^2+^] changes with time following onset of the imposed *J*_influx_ and (**B**) the resulting dependences of the steady state changes in [Ca^2+^] upon radial distances from the centre of the T-SR junction. The [Ca^2+^] across the T-SR junction falls with reducing depolarizing steps at all points in both the steady state and with time. For comparison, the *y* axis limit has been fixed at 25 μM. (**C**–**E**) The T-SR model recapitulates experimentally reported Ca^2+^ flux densities and resulting Ca^2+^ concentrations following the graded test voltage steps. Thus: (**C**) Measured cytoplasmic [Ca^2+^] (filled symbols) achieved following test voltage steps ^45^ compared with [Ca^2+^]_edge_ (open symbols) in response to Ca^2+^ flux densities, *J*_influx_ determined from reported rates of increase in [Ca^2+^], d[Ca^2+^]/d*t*, corresponding to those test voltages (abscissa) illustrated in (**A**, **B**) at exit length *λ* = 9.2 nm. (**D**) Plot of [Ca^2+^]_edge_ from the reconstructed T-SR junction against reported cytoplasmic [Ca^2+^]^45^. The points fit a linear regression model with a gradient close to 1 (1.02) and intercepts close to the origin. (**E**) Plot of Ca^2+^ influx across the SR membrane *Φ*_influx_ against computed [Ca^2+^]_edge_ expressed in SI units. The points fit a linear regression model with a gradient and zero intercept matching that expected from the conservation condition. The matching computed and observed [Ca^2+^] in (**C**) and the gradients and intercepts in (**D**) and (**E**) confirm match of the T-SR junction model to previous experimental results at different test voltages.

Firstly, all the test voltages were associated with T-SR junction Ca^2+^ microdomains (Fig. 3). Figure 3A plots diffusion equation solutions with time after imposing the Ca^2+^ influx to reach steady state [Ca^2+^] values at the centre and edge of the T-SR junction, and halfway between these points. Figure 3B shows these dependences of the steady state [Ca^2+^] with radial distance close to the T-tubular (superscript ‘T’) and SR membranes (superscript ‘SR’), and halfway along their axial distance (superscript ‘TSR’). [Ca^2+^] declined from its maximum values with radial distance from the T-SR junction centre (subscripts below: ‘centre’) towards the T-SR edge (subscript ‘edge’) with similar readouts close to the T-tubular (subscript: ‘T’) and SR membranes (subscript: ‘SR’), and half-way (subscript: ‘50%’) along their axial distance.

Secondly, quantifications of Ca^2+^ microdomains arising from the range of *J*_influx_ values and corresponding test voltages showed that these were similar in form, consistent with the linearity expected of diffusional processes. This emerged from comparing ratios between [Ca^2+^] at the T-SR centre with those at the edge and halfway between (50%) at the T-tubular and SR membranes, and within the T-SR space. Thus [Ca^2+^]^T^_50%_/[Ca^2+^]^T^_centre_, [Ca^2+^]^TSR^_50%_/[Ca^2+^]^TSR^_centre_, and [Ca^2+^]^SR^_50%_/[Ca^2+^]^SR^_centre_ values were all close to 79% and [Ca^2+^]^T^_edge_/[Ca^2+^]^T^_centre_, [Ca^2+^]^TSR^_edge_[Ca^2+^]^TSR^_centre_ and [Ca^2+^]^SR^_edge_/[ Ca^2+^]^SR^_centre_ were all close to 14%. Similarly, the radial distances from the T-SR centre over which [Ca^2+^] fell by 50% of its centre-edge range were all 77.67, 77.90 and 77.76 nm whether close to the T-tubular membrane, the SR membrane and halfway between these respectively.

Thirdly, progressively larger *J*_influx_ values corresponding to the increasing experimental d[Ca^2+^]/dt with progressive depolarization correspondingly predicted increased steady state [Ca^2+^] whether at the centre, 50% from or at the T-SR edge, or close to T-tubular or SR membranes or between these. They produced substantially higher, ~ 10’s of µM [Ca^2+^] values at the T-SR centre than the ~ µM [Ca^2+^] values at the edge.

Both the individual values of, and the relationships between, the modelled *J*_influx_ at differing test voltages and [Ca^2+^]_edge_ closely matched the corresponding reported experimental relationship between d[Ca^2+^]/d*t* and [Ca^2+^]_max_. Figure 3C shows that the voltage-matched *J*_influx_ values gave [Ca^2+^]_edge_ values similar to those of the corresponding reported experimental [Ca^2+^]_max_^45^. Figure 3D shows a linear relationship between [Ca^2+^]_edge_, and [Ca^2+^]_max_ with close to unity gradient and zero intercept. Furthermore, combining the Ca^2+^ influx and efflux equality condition, *Φ*_*influx*_ = *Φ*_efflux_, and the efflux equation (Eqs. 7–9) gives the conservation relationship:

15 $$\it \it {\Phi }_{\mathrm{e}\mathrm{f}\mathrm{f}\mathrm{l}\mathrm{u}\mathrm{x}}={J}_{\mathrm{e}\mathrm{f}\mathrm{f}\mathrm{l}\mathrm{u}\mathrm{x}}\mathrm{\pi }dw=\frac{D{\left[\mathrm{Ca}^{2+}\right]}_{\mathrm{e}\mathrm{d}\mathrm{g}\mathrm{e}}}{\mathrm{\lambda }}(\mathrm{\pi }dw)$$

This yielded data points replicating the predicted linear dependence between *Φ*_influx_ and [Ca^2+^]_edge_*.* This had a zero intercept and slope in agreement with the efflux prediction $\frac{{\Phi }_{\mathrm{e}\mathrm{f}\mathrm{f}\mathrm{l}\mathrm{u}\mathrm{x}}}{{\left[\mathrm{C}{\mathrm{a}}^{2+}\right]}_{\mathrm{e}\mathrm{d}\mathrm{g}\mathrm{e}}}=\frac{D}{\lambda }(\mathrm{\pi }dw)$ = 3.606 × 10^–17^ m^3^/s (Fig. 3E). Finally, in accord with linearity predictions, computations adopting further tenfold *J*_influx_ reductions similarly (A) converged monotonically to steady state [Ca^2+^] measurable at selected radial distances from the T-SR junction centre yielding (B) uniform radial [Ca^2+^] gradients along the T-SR distance visualized as (C) radial [Ca^2+^] gradients extending the full axial T-SR junction distance (Supplementary Table S3 and Fig. S2). The [Ca^2+^]^T^_50%_/[Ca^2+^]^T^_centre_, [Ca^2+^]^TSR^_50%_/[Ca^2+^]^TSR^_centre_, and [Ca^2+^]^SR^_50%_/[Ca^2+^]^SR^_centre_ as well as the [Ca^2+^]^T^_edge_/[Ca^2+^]^T^_centre_, [Ca^2+^]^TSR^_edge_^/^[Ca^2+^]^TSR^_centre_ and [Ca^2+^]^SR^_edge_/[Ca^2+^]^SR^_centre_ remained essentially constant (79% and 14%). Furthermore, radial distances from the T-SR centre over which [Ca^2+^] fell by 50% of its centre-edge range, whether close to the T-tubular membrane, the SR membrane and half way between these respectively remained similar (77.67, 77.90 and 77.76 nm). [Ca^2+^] levels through the T-SR junction fell proportionally with 10- and 100-fold *J*_influx_ reductions: [Ca^2+^]^T^_centre_ fell from ~ 0.43 μM to 0.043 μM and 0.0043 μM respectively, as expected from the relationship established in Fig. 3E.

### Ca^2+^ microdomains with varied Ca^2+^ diffusion coefficients

Skeletal muscle T-SR junction properties vary with physiological conditions, both within and between muscle fibres, individual muscles and muscle types. Furthermore, such surface-cytoplasmic membrane appositions also occur in and vary amongst other cell types. Nevertheless, the Ca^2+^ microdomains robustly persisted with wide variations involving previously experimentally reported Ca^2+^ diffusion constants, *D*, and T-SR junction geometries represented by their axial distances *w*, and diameters, *d*. These further computations varied each parameter in turn holding the remaining variables constant under conditions of fixed *J*_influx_.

**Figure 4:**
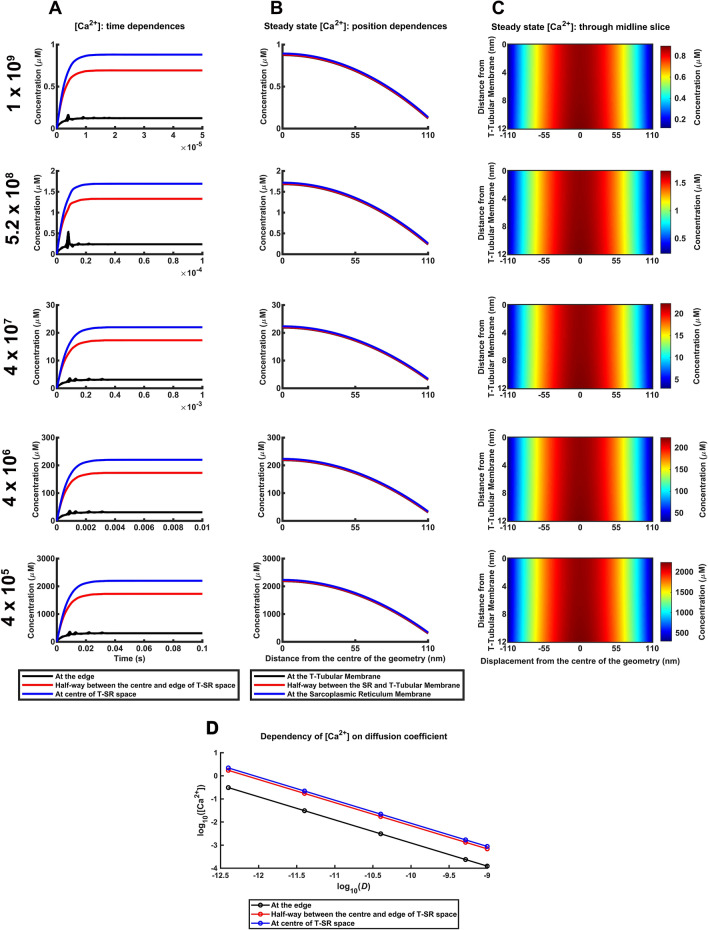
Ca^2+^ microdomain characteristics persist with variation in Ca^2+^ diffusion coefficient. (A-C) Exploring effects of variations in reported in vitro and in vivo Ca^2+^ diffusion coefficient values (units: nm^2^/s), *D*, ranging from highest reported values at infinite dilution, through two reported in vivo values, and two substantially lower values for comparison. Ca^2+^ influx density, *J*_influx*,*_ T-SR diameter, *d*, and T-SR distance, *w*, maintained constant at values adopted in the T-SR model (Table 1). (**A**) Variations in [Ca^2+^] with time following onset of imposed *J*_influx_. (**B**) Dependence of the resulting steady state [Ca^2+^] with radial distance from the centre of the T-SR junction. (**C**) Mapping of [Ca^2+^] across a midline axial slice; values of [Ca^2+^] scaled linearly with maximum [Ca^2+^] at centre of T-SR junction to illustrate features of microdomain characteristics and radial concentration gradients. (**D**) Inverse relationship between [Ca^2+^] at the centre, the edge, and halfway between these of the T-SR junction, and diffusion coefficient, *D*. Linear double logarithmic plots of [Ca^2+^]^TSR^_centre_, [Ca^2+^]^TSR^_50%_ and [Ca^2+^]^TSR^_edge_, against diffusion coefficient, *D*, coefficients corresponding to an inverse relationship between [Ca^2+^]^TSR^_centre_, [Ca^2+^]^TSR^_50%_ and [Ca^2+^]^TSR^_edge_, and *D*, at constant Ca^2+^ influx density, *J*_influx*,*_ T-SR diameter, *d*, and T-SR distance, *w*.

Firstly, we extended the modelling beyond the reported skeletal muscle cytosolic Ca^2+^ diffusion coefficient *D* = 4.0 × 10^7^ nm^2^/s^46–48^. Higher reported values reach 5.2 × 10^8^ nm^2^/s in other cell types^50^ (cf:^51–54^) and 1 × 10^9^ nm^2^s at infinite dilution in vitro^55,56^ (Supplementary Table S4). Lower *D* values might result from local concentrations of proteins, lipids or ions, suggested by the reported electron-dense T-SR junction cytosol^44^. Solutions with *D* between 4.0 × 10^5^ and 1 × 10^9^ nm^2^/s at constant *w*, *d* and *J*_influx_, continued to converge with correspondingly varied relative timescales (Fig. 4A). They predicted steady state radial [Ca^2+^] gradients extending the full axial distance between T and SR membranes (Fig. 4B,C). Radial dependences of [Ca^2+^] with distance from the T-SR centre (Fig. 4B) were not altered by the changes in *D*. Ratios between [Ca^2+^] at the edge and halfway from the centre, to [Ca^2+^] at the T-SR centre whether close to T-tubular or SR or between the two membranes, remained at 79% and 14% respectively. Distances for [Ca^2+^] to fall to 50% between centre and edge values similarly remained unchanged. However, diffusion coefficient value markedly influenced [Ca^2+^] at the centre of the T-SR junction.

Nevertheless, even the highest *D* = 1 × 10^9^ nm^2^/s free diffusion value at infinite dilution predicted near µM-[Ca^2+^] microdomains. Conversely, successive tenfold reductions in *D* down to 4.0 × 10^5^ nm^2^/s resulted in limiting ~ mM hypothetical TSR [Ca^2+^] levels approaching reported in situ free SR [Ca^2+^] (~ 3.6 mM; assuming a 6.1 mM SR [calsequestrin] with a 1.1 mM Ca^2+^ binding constant^57^). Plotting log_10_[Ca^2+^] values at the centre, edge of the T-SR junction, and halfway between these against log_10_(*D*) all yielded similar linear plots reflecting the constant radial [Ca^2+^] profiles shown by the Ca^2+^ microdomains (Fig. 4D). Their constant gradient indicated inverse [Ca^2+^]-*D* relationships and a ${\{\left[\mathrm{C}{\mathrm{a}}^{2+}\right]}_{\mathrm{e}\mathrm{d}\mathrm{g}\mathrm{e}}/(1/D)\}$ proportionality constant, 1.27 × 10^–13^ mol/(ms) agreeing with the steady state $\it {\Phi }_{\mathrm{i}\mathrm{n}\mathrm{f}\mathrm{l}\mathrm{u}\mathrm{x}}={\Phi }_{\mathrm{e}\mathrm{f}\mathrm{f}\mathrm{l}\mathrm{u}\mathrm{x}}$ conservation condition for which:

16 $${\left[\mathrm{Ca}^{2+}\right]}_{\mathrm{e}\mathrm{d}\mathrm{g}\mathrm{e}}=\left(\frac{\lambda d{J}_{\mathrm{i}\mathrm{n}\mathrm{f}\mathrm{l}\mathrm{u}\mathrm{x}}}{4wD}\right)$$

### Ca^2+^ microdomains at increased T-SR distances

**Figure 5:**
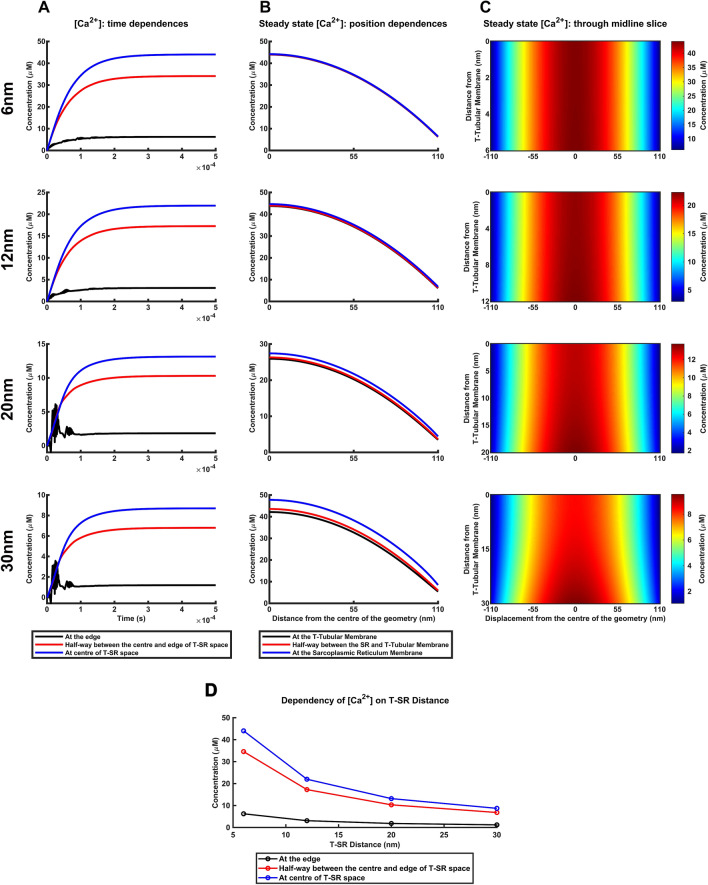
Altered Ca^2+^ microdomain characteristics with increased T-SR distances.. (**A**-**C**) The effect of variations in reported experimental values of T-SR distance, *w*, ranging from lowest reported values in hypertonic extracellular conditions to the greatest values associated with fatiguing exercise at constant imposed Ca^2+^ influx density, *J*_influx_ diffusion coefficient, *D*, and TSR diameter, *d*. (**A**) Variations in [Ca^2+^] with time following onset of imposed *J*_influx_. (**B**) Resulting variations in steady state [Ca^2+^] with radial distance from the centre of the T-SR junction; note significant differences in the [Ca^2+^] magnitudes close to the T-tubular, the sarcoplasmic reticular (SR) membranes, and the intervening T-SR region. (**C**) Mapping of [Ca^2+^] across a midline axial slice; values of [Ca^2+^] are scaled linearly with maximum concentration at centre of T-SR junction [Ca^2+^] to illustrate features of microdomain characteristics and the radial concentration gradients. Note marked pluming of the Ca^2+^ microdomain with increasing T-SR distance. (**D**) Dependence of T-SR junctional [Ca^2+^] at the centre, the edge, and halfway between these upon T-SR distance, *w*. Relationships emerging from plots of [Ca^2+^]^TSR^_centre_, [Ca^2+^]^TSR^_50%_ and [Ca^2+^]^TSR^_edge_, against T-SR distance, *w*, at constant diffusion coefficient, *D*, influx density, *J*_influx_ and T-SR diameter, *d*.

Secondly, T-SR distances also vary with physiological conditions (Supplementary Table S5). They fall from *w* = 12 nm to 6.6 nm in hypertonic extracellular solutions^58^. They increase to 20.15 nm and 29.60 nm with fatiguing low-frequency intermittent stimulation^59^ and exposure to hypotonic solutions^60^ respectively. Computational solutions modelling these variations in *w* at constant *J*_influx_, *D* and *d* (Fig. 5) showed early instabilities with time at the greatest *w* values (Fig. 5A). Nevertheless, all solutions ultimately converged to steady state Ca^2+^ microdomains with increased [Ca^2+^] at the T-SR centre declining with radial distance. However, differences between [Ca^2+^] values close to the SR, the T-tubular membranes, and within the intervening space occurred at the greater T-SR distances (Fig. 5B). The colourmaps then correspondingly showed marked axial, in addition to radial [Ca^2+^] non-uniformities (Fig. 5C) with a plume-like tapering. This contrasts with the small axial nonuniformities at *w* = 12 nm becoming even smaller with its reduction to *w* = 6.6 nm. This also directly contrasts with the previous near-uniform Ca^2+^ microdomain radial profiles through the entire T-SR junctional distance observed in the computations varying *J*_influx_ and *D*.

Quantification of these effects as *w* increased from 6 to 30 nm, showed that close to the T-tubular membrane, [Ca^2+^]^T^_50%_/[Ca^2+^]^T^_centre_ and [Ca^2+^]^T^_edge_/[Ca^2+^]^T^_centre_ remained at ~ 79% and ~ 14% respectively. Similarly at the midpoint between T-tubular and SR membranes, [Ca^2+^]^TSR^_50%_/[Ca^2+^]^TSR^_centre_ and [Ca^2+^]^TSR^_edge_/[Ca^2+^]^TSR^_centre_ were ~ 78% and ~ 13% respectively. However, close to SR membrane, [Ca^2+^]^SR^_50%_/[Ca^2+^]^SR^_centre_ and [Ca^2+^]^SR^_edge_/[Ca^2+^]^SR^_centre_ increased to ~ 80% and ~ 18% respectively. Furthermore, the axial nonuniformities in [Ca^2+^] close to the SR and T-tubular membranes involved both the centre and edge of the T-SR junction. Thus, [Ca^2+^]^SR^_centre_/[Ca^2+^]^T^_centre_ increased from 1.005 to 1.133 and [Ca^2+^]^SR^_edge_/[Ca^2+^]^T^_edge_ increased from 1.031 to 1.565.

In addition, radial distances for [Ca^2+^] to fall to 50% as a fraction of the total T-SR radius showed contrasting patterns at the T-tubular and SR membranes with increasing *w*. *X*_centre_/(T-SR radius) then fell from 0.763 to 0.748 whereas *X*_edge_/(T-SR radius) increased from 0.765 to 0.793. Finally, both steady state centre and edge [Ca^2+^], whether at the T-tubular or SR membranes, or within the T-SR junction, decreased. Thus, [Ca^2+^]^T^_centre_ and [Ca^2+^]^T^_edge_ fell from 43.965 and 6.190 to 8.428 and 1.081 respectively. Nevertheless, even the lowest values of [Ca^2+^]^T^_edge_ resulted in µM-[ Ca^2+^] changes. Finally, [Ca^2+^]_edge_ inversely depended upon *w* (Fig. 5D). The ${(\left[\mathrm{C}{\mathrm{a}}^{2+}\right]}_{\mathrm{e}\mathrm{d}\mathrm{g}\mathrm{e}}\ w)$ proportionality constant (3.57 × 10^–11^ mol/m^2^) approximated predictions from parameter values in the steady state conservation condition ${\left[\mathrm{C}{\mathrm{a}}^{2+}\right]}_{\mathrm{e}\mathrm{d}\mathrm{g}\mathrm{e}}=\left(\frac{{d\lambda }^{ }{J}_{\mathrm{i}\mathrm{n}\mathrm{f}\mathrm{l}\mathrm{u}\mathrm{x}}}{4Dw}\right),$ giving $\left(\frac{{d\lambda }^{ }{J}_{\mathrm{i}\mathrm{n}\mathrm{f}\mathrm{l}\mathrm{u}\mathrm{x}}}{4D}\right)=$ 3.79 × 10^–11^ mol/m^2^.

### Ca^2+^ microdomains at decreased T-SR diameters

**Figure 6:**
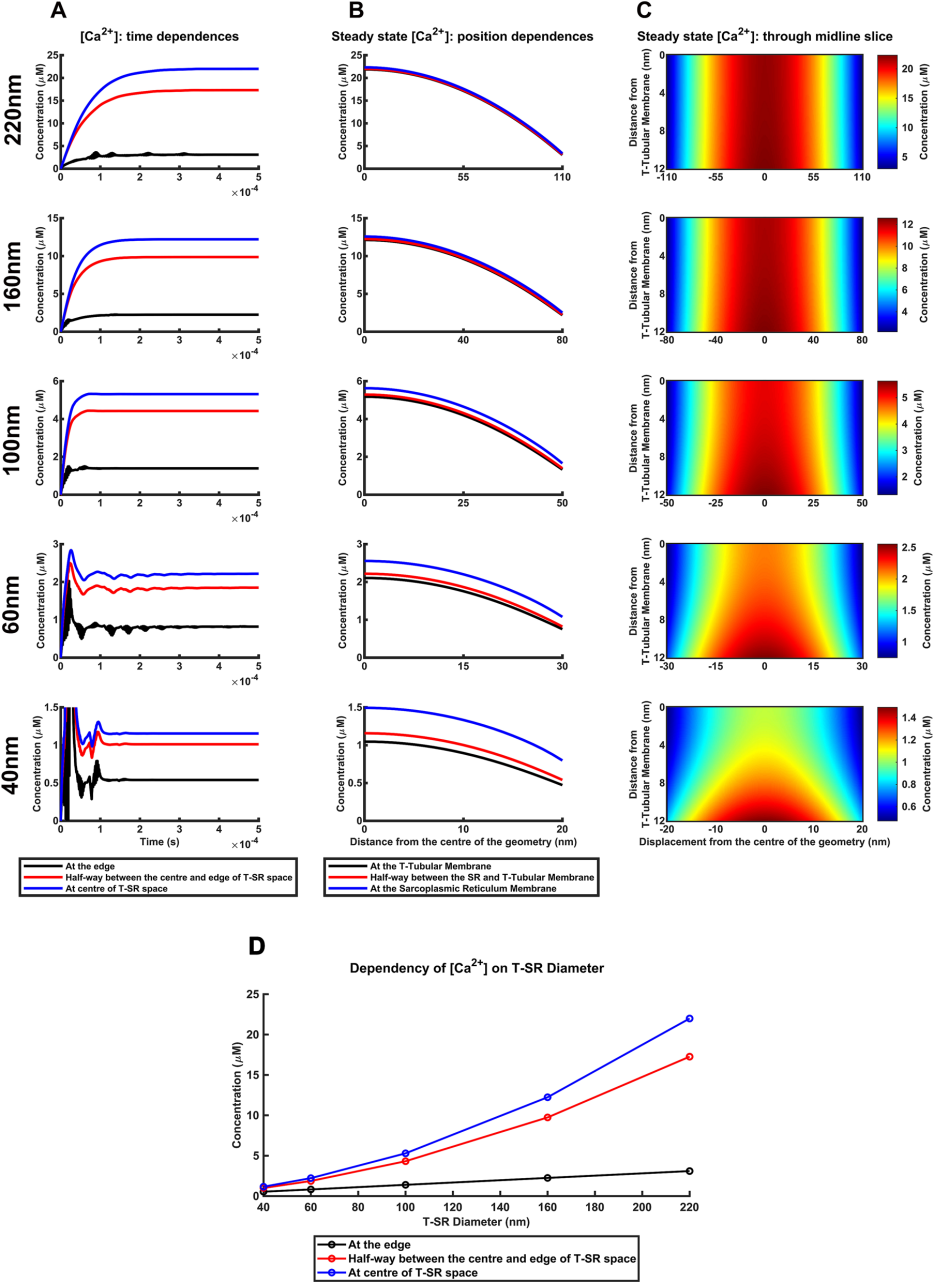
Calcium microdomain stability breaks down at decreased T-SR diameters. (**A**–**C**) Effect of reductions in T-SR diameter, *d*, from value adopted in the T-SR model. Computations at constant imposed Ca^2+^ influx density, *J*_influx_, diffusion coefficient, *D*, and T-SR distance, *w*. (**A**) Variations in [Ca^2+^] with time following onset of imposed *J*_influx_. Note initial instabilities close to the onset of imposition of *J*_influx_ at the reduced T-SR diameters. (**B**) Resulting variations in steady state [Ca^2+^] with radial distance from the centre of the T-SR junction; note significant differences in the regions close to the T-tubular and sarcoplasmic reticular (SR) membranes, and the intervening T-SR region with decreasing T-SR diameter, *d* (**C**) Mapping of [Ca^2+^] across a midline axial slice; values of [Ca^2+^] are scaled linearly with maximum concentration at centre of T-SR junction [Ca^2+^] to illustrate microdomain characteristics and radial concentration gradients. Marked pluming of Ca^2+^ microdomain with decreasing T-SR diameter. (**D**) Dependence of T-SR junctional [Ca^2+^] at the centre, the edge, and halfway between these upon T-SR diameter. Relationships emerging from plots of [Ca^2+^]^TSR^_centre_, [Ca^2+^]^TSR^_50%_ and [Ca^2+^]^TSR^_edge_, against TSR diameter, *d*, at constant diffusion coefficient, *D*, influx density, *J*_influx_ and T-SR distance *w*.

Thirdly, significant variations in effective areas of membrane appositions occur not only between skeletal muscle T-tubular and SR membrane but also occur in and between other cell types. We quantified these effects successively reducing T-SR junction diameters, *d*, from the initial 220 nm down to 40 nm, at constant *J*_influx_, *D* and *w* (Fig. 6, Supplementary Table S6). The computational solutions showed some initial instabilities but ultimately converged even at the smallest T-SR diameters (Fig. 6A). They similarly generated Ca^2+^ microdomains in which [Ca^2+^] declined with radial distance from the T-SR junction centre. These radial gradients accompanied significant axial [Ca^2+^] variations at the T-tubular and SR membranes and the intervening space (Fig. 6B) resulting in plume-like tapering at the smallest T-SR diameters in the colourmaps (Fig. 6C).

Quantifications of these alterations in Ca^2+^ microdomain characteristics first demonstrated changes in [Ca^2+^]^T^_50%_/[Ca^2+^]^T^_centre_ and [Ca^2+^]^T^_edge_/[Ca^2+^]^T^_centre_ from 78 and 14% respectively at *d* = 220 nm to 86% and 45% at *d* = 40 nm. There were also significant [Ca^2+^] differences between T-SR regions close to the T-tubular and SR membranes with [Ca^2+^]^SR^_centre_/[Ca^2+^]^T^_centre_ and [Ca^2+^]^SR^_edge_/[Ca^2+^]^T^_edge_ at 1.021 and 1.111 at *d* = 220 nm to 1.426 and 1.689 at *d* = 40 nm. The distances through which [Ca^2+^] fell to half of its maximum value as a proportion of T-SR radius (*X*_centre_/(T-SR radius)) and (*X*_edge_/(T-SR radius)) increased from 0.761 to 0.768 and 0.950 to 1.000 respectively. Finally, [Ca^2+^]^T^_centre_ and [Ca^2+^]^T^_edge_ fell from 21.876 and 3.025 to 1.047 and 0.473 respectively. Thus, even a *d* = 40 nm diameter produced µM-[Ca^2+^] microdomain differences. [Ca^2+^]_edge_ positively correlated with *d*, with slopes between 1.41 × 10^4^ to 1.35 × 10^4^ mol/m^4^ in agreement with steady state flux conservation, giving ${\left[\mathrm{C}{\mathrm{a}}^{2+}\right]}_{\mathrm{e}\mathrm{d}\mathrm{g}\mathrm{e}}=\frac{\lambda {dJ}_{\mathrm{i}\mathrm{n}\mathrm{f}\mathrm{l}\mathrm{u}\mathrm{x}}}{4Dw}$ for which $\frac{\lambda {J}_{\mathrm{i}\mathrm{n}\mathrm{f}\mathrm{l}\mathrm{u}\mathrm{x}}}{4Dw}=$ 1.44 × 10^4^ mol/m^4^ (Fig. 6D).

### Ca^2+^ microdomains in resting muscle fibres

**Figure 7:**
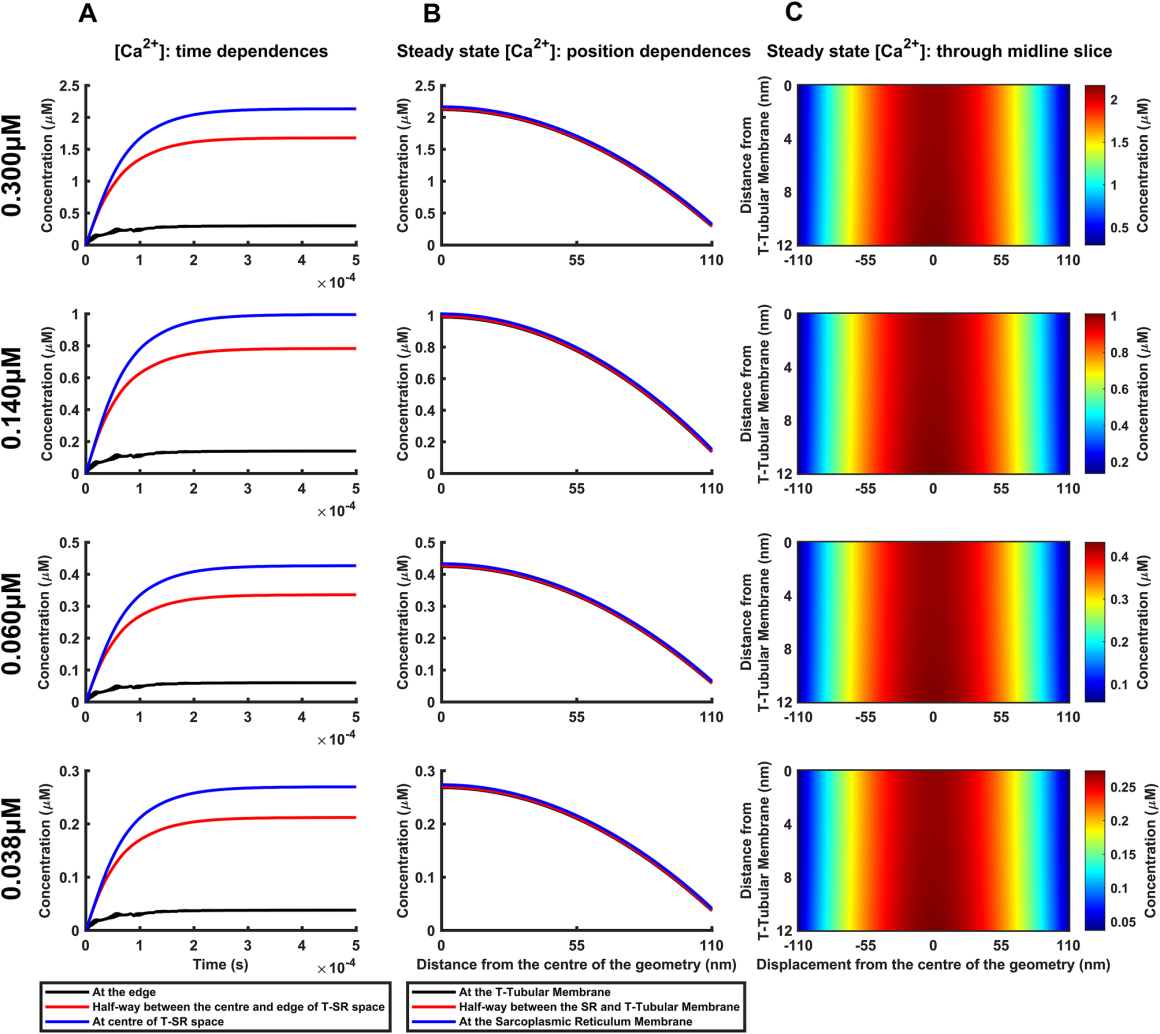
Ca^2+^ microdomains attain micromolar [Ca^2+^] levels at edge [Ca^2+^] corresponding to resting [Ca^2+^]. Effects of variations in Ca^2+^ influx density, *﻿J*_influx,_ generating T-SR junction edge Ca^2+^ concentrations, [Ca^2+^]_edge_ achieving previously reported resting [Ca^2+^] levels studied at constant values of diffusion coefficient, *﻿D*, T-SR diameter, *d﻿*, and distance, *﻿w*. (**A**) Variations in [Ca^2+^] with time following onset of imposed *﻿J*_influx_. (**B**) Resulting variations in steady state [Ca^2+^] with radial distance from the centre of the T-SR junction. (**C**) Mapping of [Ca^2+^] across a midline axial slice; values of [Ca^2+^] are scaled linearly with maximum concentration at centre of T-SR junction [Ca^2+^] to illustrate persistence and features of microdomains including their radial concentration gradients.

Finally, the recent reports that had suggested an existence of T-SR junction Ca^2+^ microdomains included evidence suggesting background ryanodine receptor (RyR)-mediated Ca^2+^ fluxes modulating Na_v_1.4 function even in resting skeletal muscle^27,30^. Available Fura-2 studies suggest resting cytosolic [Ca^2+^] between 0.06 and 0.14 μM^61^. The highest reported, Fluo-3, studies reported up to 0.30 μM^62,63^ and the lowest Ca^2+^-sensitive microelectrode measurements, 0.038 μM^64^ (Supplementary Table S7). These reports permitted determination of values for the corresponding background *J*_influx_ from the influx and efflux equations for *Φ*_influx_ and *Φ*_efflux_ respectively, assuming the conservation condition *Φ*_influx_ = *Φ*_efflux_. Approximating these [Ca^2+^] values to ${\left[\mathrm{C}{\mathrm{a}}^{2+}\right]}_{\mathrm{e}\mathrm{d}\mathrm{g}\mathrm{e}}$ would give the required ${J}_{\mathrm{i}\mathrm{n}\mathrm{f}\mathrm{l}\mathrm{u}\mathrm{x}}=\frac{4Dw{\left[\mathrm{C}{\mathrm{a}}^{2+}\right]}_{\mathrm{e}\mathrm{d}\mathrm{g}\mathrm{e}}}{\lambda d}$*.* Employing this resting myoplasmic [Ca^2+^] range (Fig. 7A) indicated the existence of Ca^2+^ microdomains with radial concentration profiles (Fig. 7B,C), ratios of [Ca^2+^] at the edge and halfway between edge and centre, and the centre (79% and 14%), and *X’*_centre_, *X’*_50%_, and *X’*_rim_ values (77.67, 77.76 and 77.90 nm) all identical to corresponding values at higher *J*_influx_. Finally, the [Ca^2+^]^T^_centre,_, [Ca^2+^]^TSR^_centre_ and [Ca^2+^]^SR^_centre_ approached 1 µM [Ca^2+^] concentrations, giving 0.268, 0.270 and 0.274 at the lowest and 0.990, 0.995 and 1.010 µM at the highest resting [Ca^2+^].

### Physiological implications of modelled T-SR junction Ca^2+^ microdomains

**Figure 8:**
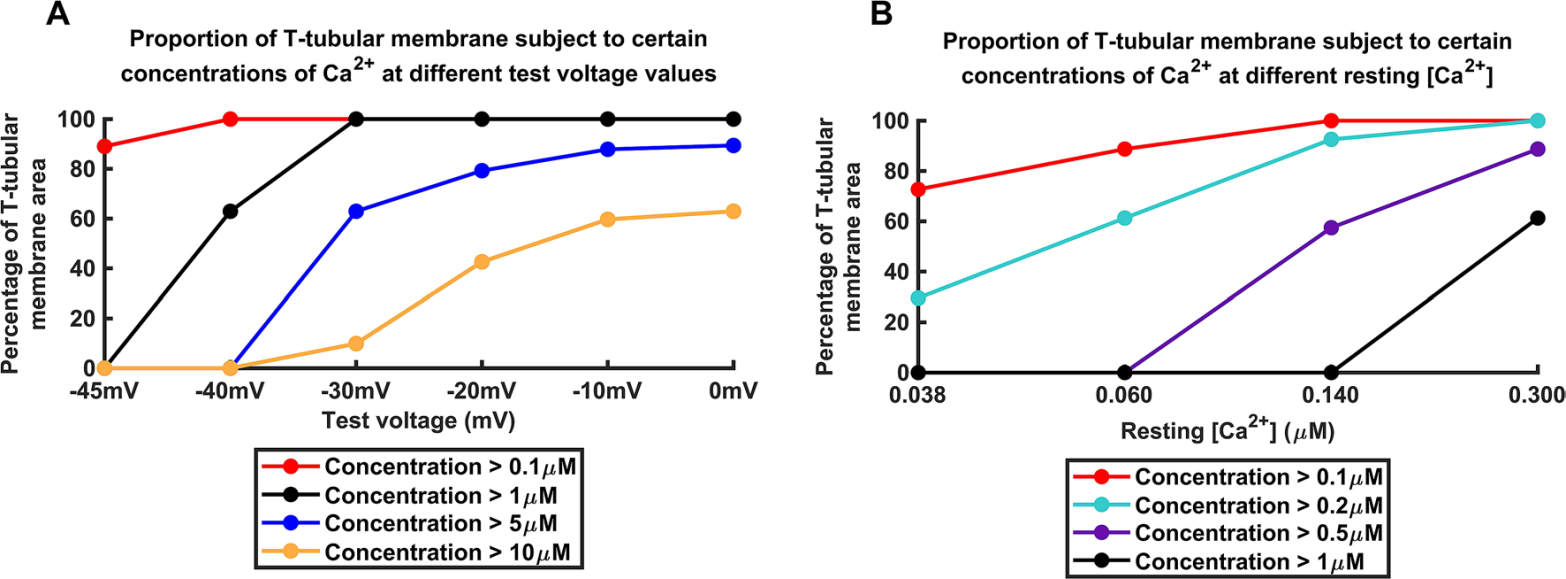
Proportions of T-SR junction T-tubular membrane area exposed to varied tested microdomain [Ca^2+^]. Results shown for (**A**) active muscle at different test voltages (Fig. 3) and (**B**) resting (Fig. 7) skeletal muscle using previously modelled resting [Ca^2+^] values.

Together, the present findings substantiate recent experimental reports predicting physiologically significant T-SR Ca^2+^ microdomains in skeletal muscle. These had implicated RyR-mediated SR Ca^2+^ release in modulating tubular Na_v﻿_1.4 function in both activated and resting skeletal muscle^27,30^. The spatial [Ca^2+^] data arrays obtained from the model of activated (Fig. 3; Table 1) and resting muscle (Fig. 7) were used to derive the respective proportions of T-tubular membrane facing the T-SR junction model exposed to different microdomain [Ca^2+^] (Fig. 8). In activated muscle, successively greater proportions of such T-tubular membrane became exposed to successively higher, 0.1 to 10 µM [Ca^2+^] with increasing depolarization (Fig. 8A). The latter included exposures to concentrations exceeding the 1.0 µM [Ca^2+^] levels corresponding to dissociation constants, *K*_d_ of typical modulatory proteins including calmodulin. Furthermore, the tested values of resting [Ca^2+^] similarly predicted exposures of significant proportions of T-tubular membrane to lower, but nevertheless significant, 0.5 µM [Ca^2+^] (Fig. 8B).

## Discussion

Recent experimental reports implicated hypothetical Ca^2+^ microdomains in paradoxical Ca^2+^-mediated effects on skeletal muscle Na_v﻿_1.4 activation following pharmacological manoeuvres increasing or decreasing RyR-mediated SR Ca^2+^ release^26,27,30^. They went on to suggest that T-SR junctional sites of close membrane proximity, key to excitation contraction coupling, might form diffusion-restricted, ultrastructurally dispersed intracellular subcompartments. Although accounting for only a small proportion of total cytosolic volume, these might sustain regulatory local [Ca^2+^] heterogeneities in the vicinity of the tubular Na_v﻿_1.4^35–37^. These could potentially cause local ~ μM-[Ca^2+^] alterations previously reported to modify Na^+^ channel function^23^ arising from direct Ca^2+^, or indirect, Ca^2+^-calmodulin mediated, binding to Na_v﻿_1.4^18–20,23,24^. Our present modelling studies accordingly explored and characterized conditions required for such Ca^2+^ microdomain formation within these T-SR junctional structures.

*The structural parameters describing sarcomere, surface, T-tubular and SR membrane structure, and distributions, densities and electron microscope ultrastructure of their T-SR junctional regions required for such modelling were available for amphibian skeletal muscle*^11–13,39–44^. [Ca^2+^] gradients through the resulting formalized geometrical model of a typical T-SR junction in both resting and stimulated muscle fibres were then resolved by finite element method (FEM) solutions of basic Fick diffusion equations. Their boundary conditions first included a RyR-mediated Ca^2+^ release into the T-SR space by a uniform Ca^2+^ influx density *J*_influx_ across its SR face. Subsequent Ca^2+^ diffusion with a diffusion coefficient established from previous experimental reports from amphibian myoplasm was then mapped through the radially symmetric T-SR junctional space. The second, efflux, boundary condition at the edge of the modelled junction was similarly described by a first order [Ca^2+^]-dependent process into a well-stirred cytosolic space of infinitely large volume. The latter formalism further matched previous reported eventual first order steady state SERCA-mediated resequestration of the released cytosolic Ca^2+^^49^.

The boundary conditions simulated conditions both of full and of graded activation by previously reported experimental voltage clamp steps from resting to both 0 mV and varying test potentials and the resulting alterations in cytosolic [Ca^2+^]^45^. At the influx boundary, Ca^2+^ influx densities *J*_influx_ for each voltage were determined directly from the corresponding reported maximum rates of SR Ca^2+^ release, d[Ca^2+^]/d*t*, and the model geometrical properties. At the efflux boundary, the resulting [Ca^2+^] at the edge of the T-SR junction, [Ca^2+^]_edge_, was first matched to the corresponding experimental maximum cytosolic concentration [Ca^2+^]_max_, by optimising the single free parameter giving exit length *λ* = 9.2 nm under conditions of full activation by a test step to 0 mV. This assumed the quantities were proportional and close to each other. Both the latter approximations were then further tested in subsequent explorations of varying *J*_influx_ through different test voltages. In all events, further corrections for any discrepancies arising from a small [Ca^2+^]_edge_ > [Ca^2+^]_max_ would tend to enhance rather than reduce the computed [Ca^2+^] magnitudes.

*The resulting T-SR model predicted *Ca^2+^ *microdomains fulfilling the previous suggestions*^30^. The computational solutions following step impositions of *J*_influx_ converged to give steady state T-SR junctional Ca^2+^ microdomains. Their heatmap representations demonstrated radial [Ca^2+^] gradients extending the entire axial T-SR junction distance. The microdomains were quantified radially at the centre, at distances halfway to, and at the edge of the T-SR junction, in axial regions close to the T-tubular and SR membranes and in the intervening T-SR junction space. Furthermore, varying *J*_influx_ to reflect previously experimentally reported d[Ca^2+^]/d*t*, obtained at varying test voltages, at a constant *λ* value, gave predicted voltage dependences of [Ca^2+^]_edge_ closely approximating those of the corresponding experimental [Ca^2+^]_max_. The accordingly linear, [Ca^2+^]_edge_–[Ca^2+^]_max_ relationship had unity gradient and zero intercept. Furthermore, the [Ca^2+^]_edge_ values themselves depended linearly on the corresponding Ca^2+^ influx *Φ*_influx_ terms with a gradient fulfilling predictions from the geometrical terms in the equation for the corresponding Ca^2+^ efflux.

All these *J*_influx_ conditions consistently generated T-SR junction Ca^2+^ microdomains containing physiologically significant, ~ μM-[Ca^2+^], heterogeneities graded with imposed depolarization. These could locally elevate [Ca^2+^] from ~ 0.3–0.4 µM at activation threshold, to 17–20 µM at full activation, relative to the remaining bulk extra-junctional cytosolic [Ca^2+^]. These microdomains all extended through the entire axial T-SR distance. They showed similar normalised [Ca^2+^] profiles with radial distance, in which [Ca^2+^] declined > fivefold from its maximum in the centre to the edge of the T-SR junction. The spatial [Ca^2+^] dependences persisted with proportionately reduced [Ca^2+^] even with 10 and 100-fold *J*_influx_ reductions below threshold levels for observed Ca^2+^ release^45^. These modelling studies adopting established quantifications for baseline T-SR and sarcomere membrane structure, and diffusion coefficient values, *D* could thus replicate reported physiological d[Ca^2+^]/d*t* and [Ca^2+^]_max_ in activated amphibian skeletal muscle.

Detailed characteristics of such membrane appositions vary amongst muscle or cell types, and with physiological and physical conditions. Nevertheless, *microdomain formation and characteristics were robust through systematic tests at constant J*_*influx*_* that varied *Ca^2+^ *diffusion coefficient, D, T-SR distance, w, and T-SR diameter, d, in turn, holding the remaining variables constant.* These tests further made it possible to survey the relative importance of diffusional or geometric properties to microdomain formation and characteristics.

First, alterations in *D* within the T-SR space could reflect Ca^2+^ buffering capacities, κ = (Δ[bound Ca]/Δ[free Ca])), of its contained immobile and mobile buffers, and the diffusion coefficient *D*_mobile_ of the mobile buffer. Assuming the rapid buffer approximation, the resulting steady state *D* is related to the free diffusion coefficient *D*_Ca2+_ by the expression^65–67^:

$${D}_{\mathrm{C}\mathrm{a}2+}\left\{1+ \frac{{D}_{\mathrm{m}\mathrm{o}\mathrm{b}\mathrm{i}\mathrm{l}\mathrm{e}}}{{D}_{\mathrm{C}\mathrm{a}2+}}\right\}{\kappa }_{\mathrm{m}\mathrm{o}\mathrm{b}\mathrm{i}\mathrm{l}\mathrm{e}}/\left\{1+{\kappa }_{\mathrm{m}\mathrm{o}\mathrm{b}\mathrm{i}\mathrm{l}\mathrm{e}}+{\kappa }_{\mathrm{i}\mathrm{m}\mathrm{m}\mathrm{o}\mathrm{b}\mathrm{i}\mathrm{l}\mathrm{e}}\right\}$$

*Immobile buffer* could reflect fixed Ca^2+^ binding sites including negatively charged membrane bilayer phospholipid groups^68^ and Ca^2+^-binding domains in Ca^2+^ dependent ion channel, cytoskeletal, transport motor and membrane-associated Ca^2+^ binding kinase proteins^69^. This would generate local, steady state equilibria between Ca^2+^ binding and Ca^2+^ diffusion: depleted Ca^2+^-free immobile buffer cannot be replaced by diffusion from remote sites. Immobile buffer would then leave steady-state Ca^2+^ microdomains unaffected^67^. Whilst its action could alter the [Ca^2+^] kinetics, our modelled step impositions of Ca^2+^ influxes increased T-SR free [Ca^2+^] to steady state values over ~ 0.2 ms exponential timecourses. These were 1–2 orders of magnitude shorter than those of experimentally observed Ca^2+^ transients^45,70^. In contrast, *mobile buffer* could influence *D* to extents dependent upon ${\kappa }_{\mathrm{m}\mathrm{o}\mathrm{b}\mathrm{i}\mathrm{l}\mathrm{e}}$ and *D*_mobile_. Our computations explored a wide range of conditions extending from limiting maximal *D* values at infinite Ca^2+^ dilution without buffer^55^ then progressively reducing *D* to values yielding T-SR [Ca^2+^] values approaching and exceeding SR [Ca^2+^]. They included physiologically realistic values corresponding to known in vivo Ca^2+^ buffering capacities of ~50 and ~100 in the respective absence and presence of 1 mM MgATP^51,71^, and empirical in vivo skeletal muscle values^46–48^.

These explorations demonstrated persistent [Ca^2+^] heterogeneities despite large increases or decreases in diffusion coefficient *D* relative to established skeletal myocyte cytosolic values (*D* = 4.0 × 10^7^ nm^2^/s)^46–48^, including values reported either in vitro^55,56^ or in other cell types^50–54^. On the one hand, even the highest reported, in vitro, *D* = 1 × 10^9^ nm^2^/s, value corresponding to infinite dilution^55^ persistently yielded [Ca^2+^] microdomains approaching μM-[Ca^2+^] at the T-SR junction centre. On the other, the electron-densities within T-SR junctions could reflect local protein, lipid or ion concentrations reducing *D*^44^. Here, progressive tenfold decreases down to *D* = 4.0 × 10^5^ nm^2^/s predicted ~ mM hypothetical [Ca^2+^] actually approaching reported in situ free SR [Ca^2+^] (~ 3.6 mM; assuming a 6.1 mM SR [calsequestrin] with a 1.1 mM Ca^2+^ binding constant^57^). These correspondingly slowed the formation time courses of such microdomains. Nevertheless, in the steady state, through this entire explored *D* range, Ca^2+^ microdomains persisted with unchanged radial [Ca^2+^] profiles extending the full distance between T and SR membranes confirming the linearity condition in this model system. [Ca^2+^]_edge_ varied inversely with *D*, giving a dependence and proportionality constant that matched predictions of the T-SR junctional model.

Secondly, whilst averaging *w* = 12 nm in resting muscle, axial T-SR distances range from 6 nm with increased extracellular tonicity^58^ to 20 nm with fatiguing stimulation^59,60^. Varying *w* through this range here disrupted microdomain characteristics resulting in heatmaps showing tapering plume-like appearances at the largest T-SR distances. There were increased radial [Ca^2+^] nonuniformities themselves varying along the axial distance between SR and T-tubular membranes, to extents increasing with increasing *w*. Nevertheless, [Ca^2+^]_edge_ varied with *w*, through an inverse relationship with proportionality constant matching predictions of the T-SR junction model. Thirdly, successive reductions of T-SR junctional diameters, from *d* = 220 nm to *d* = 40 nm, similarly disrupted Ca^2+^ microdomain heatmaps again giving tapering plume-like forms at the smallest diameters. These were quantified as increased radial non-uniformities and marked axial [Ca^2+^] differences between regions close to the SR and T-tubular membranes and the intervening space along the axial T-SR distance. Falls in [Ca^2+^] with radial distance from the T-SR junction centre and [Ca^2+^] at the SR relative to the T-tubular membranes became less marked with decreasing *d*. Finally [Ca^2+^]_edge_ increased with *d* as expected from the T-SR junction model. Nevertheless, through both these latter modifications in T-SR junction geometry, despite their altered spatial characteristics, the ~ μM-[Ca^2+^] heterogeneities between their periphery and centre persisted even with more than 100% increases in T-SR distance or 75% reductions in T-SR diameter from control values derived from established morphometric data.

The previous experimental reports had also invoked background, RyR-mediated influxes of Ca^2+^ in Ca^2+^ microdomain generation in resting in addition to activated muscle^27,30^. Accordingly, *the T-SR junction model was extended further to investigate formation and properties of *Ca^2+^ *microdomains in resting myocytes*. This employed background *J*_influx_ values calculated from previously reported cytosolic [Ca^2+^] values in resting muscle. These previous Fura-red fluorescence studies had suggested experimental resting [Ca^2+^] ranging between 0.060 to 0.140 μmol/dm^3^ (ref. ^61^). We further explored further reduced resting [Ca^2+^] limits of ~ 0.038 μmol/dm^3^ from Ca^2+^-sensitive microelectrode studies^64^ and possible higher 0.300 μmol/dm^3^ limits^62,63^. The resulting T-SR modelling continued to predict Ca^2+^ microdomains with their characteristic spatial characteristics. Furthermore, microdomain [Ca^2+^] levels at the T-SR centre, whether close to the T-tubular or SR membranes or in the intervening space, approached or attained ~ μM-[Ca^2+^].

The present findings taken together could be used to reconstruct the proportions of T-tubular membrane area and therefore of resident Na_v﻿_1.4 exposed to successively greater levels of T-SR junction microdomain [Ca^2+^] in both activated and resting muscle. Successively greater proportions of activated T-tubular membrane became exposed to successively higher, 0.1 to 10 µM [Ca^2+^] with increasing depolarization. In addition, significant proportions of even resting T-tubular membrane remained exposed to significant, ~ 0.5 µM [Ca^2+^]. These findings therefore provide a physical basis for the previous suggestions implicating Ca^2+^ microdomain formation in observed modifications in Na_v﻿_1.4 function^27,30^. Ca^2+^-CaM binding takes place with ~ µM [Ca^2+^] dissociation constants^72,73^. Feedback µM-[Ca^2+^] levels arising from SR Ca^2+^ release could therefore potentially modify both skeletal^26,27,30^ and cardiac muscle^28,29^ Na_v﻿_1.4 or Na_v﻿_1.5 through direct or indirect, Ca^2+^-calmodulin (Ca^2+^-CaM) mediated, actions on their C-terminal domains^16,18–20,23,24^. Such concentrations further match the photorelease-induced 1–2 µM cytosolic [Ca^2+^] elevations previously reported to modify Na_v﻿_1.4 function^23^.

In skeletal muscle, elevated T-SR junctional microdomain [Ca^2+^] could inhibit tubular Na_v_1.4 function following normal sustained activity^59,60^ and contribute to particular clinical skeletal myopathies^74^. A myotonic hyperexcitability disorder disrupting Ca^2+^-mediated inhibition of Na_v_1.4 function has been associated with Na_v_1.4 C-terminal EF hand-like domain mutations^75,76^. A condition associated with increased myotube diameters and resting [Ca^2+^]_i_, and decreased RyR1-mediated Ca^2+^ release reflecting possible abnormalities in triad junction formation and maintenance is associated with another, junctophilin (JP2), mutation^77^. In murine cardiac muscle, Na_v﻿_1.5 inhibition followed increased SR Ca^2+^ release following pharmacological challenge^28,29^ and in RyR2-P2328S variants modelling the pro-arrhythmogenic catecholaminergic polymorphic ventricular tachycardia^78–80^. In these examples, the underlying in vivo source of microdomain Ca^2+^ would likely remain the RyR-mediated Ca^2+^ release modelled here, as opposed to T-tubular Ca_v﻿_1.1 or Ca_v﻿_1.2 channel mediated entry of extracellular Ca^2+^. Thus, early Ca^2+^ skeletal muscle voltage clamp currents, *I*_Caf_ (~ 25 µA/cm^2^)^81^ and later cell attached patch clamped cardiomyocyte *I*_CaL_ (~ 10 pA/pF^82^; would contribute *J*_influx_ terms (~ 8.64 × 10^–7^ and ~ 6.91 × 10^–8^ mol/m^2^/s assuming similar *C*_T_/*C*_S_ and *ξ*; respectively) one and two orders of magnitude lower than the corresponding RyR-mediated *J*_influx_, at comparable test voltages. The larger skeletal muscle late *I*_Ca_ (80 µA/cm2, giving *J*_influx_ ~ 2.76 × 10^–6^ mol/m^2^/s) evolves over time courses (hundreds of ms) more prolonged than excitation contraction coupling and shows rapid off kinetics on action potential repolarization^83,84^.

Microdomain µM-[ Ca^2+^] could also regulate other signalling biomolecules. They are involved in a bell-shaped in vitro open probability relationship for single channel RyR activation and inhibition^14^. Here, cardiac and neuronal, RyR2 and RyR3 are more Ca^2+^-sensitive than RyR1 but all are activated over the ~ 1 µM [Ca^2+^] predicted in the present analysis^85,86^. However, under their respective in vivo physiological [ATP] and [Mg^2+^] conditions, cardiac^87^ but not skeletal muscle^88^ RyR activation involves Ca^2+^-induced Ca^2+^ release. Skeletal muscle RyR activation instead involves direct allosteric coupling with either T-tubular Ca_v﻿_1.1-L-type Ca^2+^ channel voltage sensors^15^ or possibly other adjacent SR RyRs themselves coupled to such Ca_v﻿_1.1^89,90^. ~ µM Ca^2+^-CaM may also exert other cytosolic effects as on glyceraldehyde 3-phosphate dehydrogenase^91^ or itself provide local signaling domains^36^.

Closely apposed membranes potentially mediating localized Ca^2+^ signalling involving Ca^2+^-dependent proteins that would similarly permit divergent signalling at different sites also occur in widespread other cell types^9,10^. At smooth muscle SR-plasma membrane appositions^92^, local Ca^2+^ could modulate repolarizing Ca^2+^-activated K^+^ channel activity^93^. They also occur in neurons^6^; cerebellar Purkinje and hippocampal neurones similarly signal using RyR- Ca^2+^ release channels^1–4^. Amongst non-excitable cells, multiple 20–30 nm diameter membrane invaginations in thrombocyte open canalicular systems (OCS)^7,8^ form vacuolar structures apposed to membranes of the Ca^2+^-storing deep tubular system (DTS) previously compared with muscle T-SR junctions^94^. These gate inositol trisphosphate receptor rather than RyR mediated Ca^2+^ fluxes into the junction upon agonist stimulation. The theoretical analysis here thus combined available experimental anatomical and physiological data and diffusion theory to predict significant [Ca^2+^] heterogeneities in the skeletal muscle T-SR-junction. Its findings might next prompt investigations of structure and function in these further examples. These might be complemented by direct experimental fluorescent Ca^2+^ indicator [Ca^2+^]_TSR_ measurements^35–37^ were these to be able to address the small dimensions and dispersed nature of the microcompartments concerned.

## Materials and methods

### Finite element analysis

Diffusional mechanics and its consequent temporal and spatial [Ca^2+^] patterns were computationally examined in a model T-SR junction. This involved replicating the geometry within and through which the diffusional processes occurred, pre-processing through meshing and definition of physical conditions including loads, initial and boundary conditions, generation of system solutions and post-processing of the results (Supplementary Fig. S1). The matrix-based programming platform and language MATLAB (version R2020b win64 9.9.0.1467703, version released August 26th, 2020, MathWorks, Cambridge, UK) performed the required data array manipulations and generated all the graphics in Figs. 1, 2, 3, 4, 5, 6, 7, and 8 (https://www.mathworks.com/discovery/what-is-matlab.html). It was implemented on an IBM compatible computer (CPU: Intel Core; i7-4790 K CPU: 4.40 GHz (4 cores); GPU: ASUS STRIX GeForce GTX970; installed RAM: 16 GB, running Windows (Microsoft, Washington, USA) 10, Home 64-bit version 1909).

The underlying T-SR junction geometry was reconstructed virtually for use in a finite element analysis solving partial differential equations (PDEs) for the resulting diffusional processes with their accompanying boundary conditions (BCs) (See Supplementary File for software archive). The finite element method (FEM) described the complex geometry as a collection of subdomains (elements) by superimposing upon this geometry a mesh of tetrahedral elements joined at their vertices (nodes) and edges. The subdivision accurately represents this complex geometry, permits inclusion of dissimilar material properties, and provides a straightforward representation of the total solution whilst capturing local, microdomain, effects.

### Boundary conditions and equation solutions

Specified BCs provided values of the field and related variables, in the present case, the normal derivatives of the field variable in the form of a Neumann-type BC. The FEM equations are formulated such that at the nodal connections, the value of the field variable at any connection is the same for each element connected to the node. Solutions at the edges of each adjacent element are therefore equal, ensuring continuity of field variables between elements, avoiding physically unacceptable gaps or voids in the solution^38^. The original PDE problem is accordingly represented within each element with simpler equations approximating the solution to the original equations. Stationary linear problems whose coefficients are independent of the solution or its gradient yield a linear system of equations. In this case our PDE is time-dependent and hence the system of simpler equations is a set of ordinary differential equations (ODEs) then passed onto MATLAB solvers for numerical integration for solution. The FEM approximates the solution by minimizing the associated error function, automatically finding the linear combination of basis functions closest to the solution *u*. The FEM could therefore capture both concentration differences local to the T-tubular and SR membranes, and across the entire modelled geometry.

### Partial differential equation toolbox™

Meshing and application of the FEM used Partial Differential Equation Toolbox (version 3.5 installed on 8th October 2020 by MathWorks) within MATLAB. This provides functions for solving structural mechanics, heat transfer and general PDEs using the FEM (https://www.mathworks.com/products/pde.html). PDE Toolbox also provides the ability to automatically mesh the T-SR junction geometry, providing a basis for solving the diffusion PDE, and stores the solution as matrices amenable to various methods of presentation and post-processing of data within MATLAB. The PDE toolbox is designed to solve equations of the form:

17 $$m\frac{{\mathrm{d}}^{2}u}{\mathrm{d}{t}^{2}}+b\frac{\mathrm{d}u}{\mathrm{d}t}-\nabla \cdot \left(c\nabla u\right)+hu=f$$

with a generalised Neumann boundary condition of:

18 $$\overrightarrow{n}\cdot \left(c\nabla u\right)+qu=g$$

where the coefficients *m*, *b*, *h*, *f* and *g* can be functions of spatial position, the solution *u*, or its spatial gradient. In a diffusive system, this generalised problem reduces to the first order equation:

19 $$b \frac{\partial u}{\partial t}- \nabla \cdot (c \nabla u)+hu=f$$

with *b* set to unity, where *c* represents the diffusion coefficient *D*, and *h* represents the boundary flux term for the Neumann condition (compare Eq. 4).

### Ethical approval

This entirely theoretical study did not involve animal procedures.

## Supplementary Information

Supplementary Information.

## Data Availability

The data that support the findings of this study are available from the corresponding author upon reasonable request and are furthermore summarized in the Supplementary file.
