# Supplementary material for: Finite element analysis predicts Ca2+ microdomains within tubular-sarcoplasmic reticular junctions of amphibian skeletal muscle
Source: Sci Rep. 2021 Jul 13;11:14376. doi: 10.1038/s41598-021-93083-1 (PMC8277803; doi:10.1038/s41598-021-93083-1)
Supplement: Supplementary file 1 — Supplementary Information. [file 41598_2021_93083_MOESM1_ESM.docx]

| ***Supplementary Figures, Tables and Software Archive.*** |  |
| --- | --- |
| Figure S1. Computational processing scheme summarized in Methods  ***Tables summarizing computational solutions****.*  Supplementary Table S1. Characterization of model T-SR junction Ca^2+^ microdomain  Supplementary Table S2: T-SR junction Ca^2+^ microdomain properties at different test voltages  Supplementary Table S3: T-SR junction Ca^2+^ microdomains at sub-threshold Ca^2+^ flux densities  Supplementary Figure S2. Properties of Ca^2+^ microdomains at reduced T-SR junction Ca^2+^ influx densities*.*  Supplementary Table S4: T-SR junction Ca^2+^ microdomains produced with varying diffusion coefficients  Supplementary Table S5: Ca^2+^ microdomains at varied T-SR junction axial distances  Supplementary Table S6: Ca^2+^ microdomains at varied T-SR junction diameters  Supplementary Table S7: T-SR junction Ca^2+^ microdomains in resting muscle fibres | 2  3-17  4  6  8  10  11  13  15  17 |
| ***Appendix for MATLAB Code***  Parameter Specification  1) General Parameters  2) Edge Boundary Condition Options  Model Set-Up  1) Specifying Geometry  2) Meshing  3) Boundary Conditions  4) Initial Conditions  5) Partial Differential Equation Coefficients  Solution  1) Time for Solution  2) Generating Solution  3) Solution Conversion  4) Saving the Solution to File  Data Presentation  1) General Heat Map  2) Heat Map top and bottom views  3) Concentration vs time for a node: Obtain and plot value of Concentration at a specific point  4) Concentration vs radial distance  5) 3D Plot of Concentration at the T-Tubular Membrane  6) 2D Radial colormap  Calculate Calcium Flux  Function for F3 Flux | 19-26  19  19  19  19  19  20  20  20  20  20  20  20  20  21  21  21  21  22  23  24  25  25  26 |
| ***List of abbreviations***  ***References cited in supplementary file*** | 27  28 |


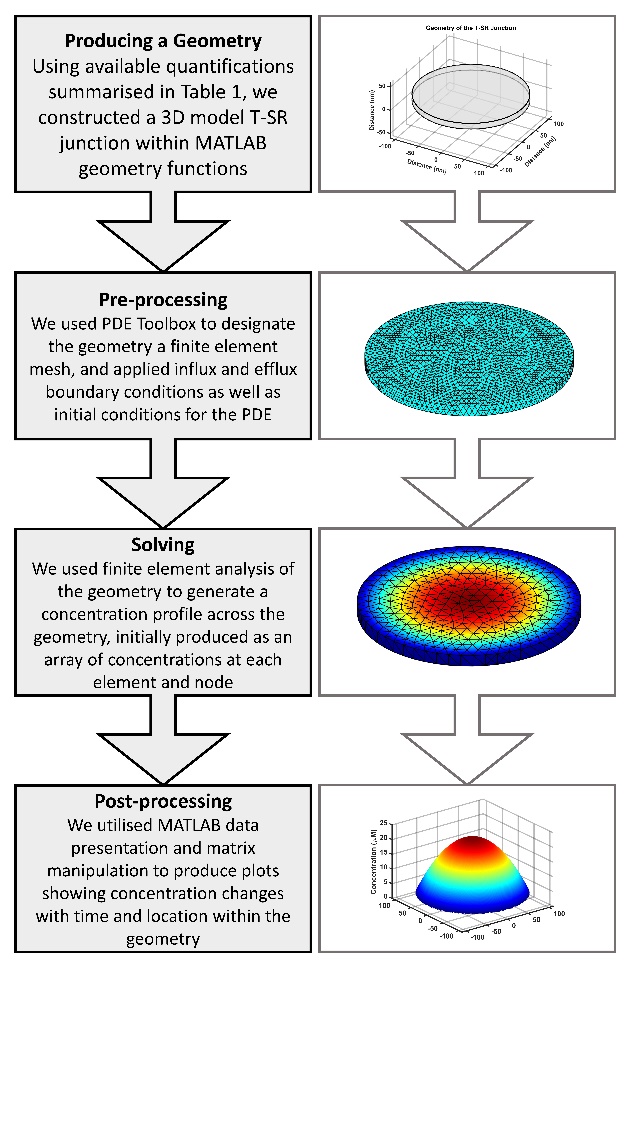
*Supplementary Figures, Tables and Software Archive.*

***Figure S1. Computational processing scheme summarized in Methods.***

Production and processing of results using the finite element method (FEM) involving a multi-step approach with each modelling run, whereby the geometry was produced, pre-processed, the partial differential equation (PDE) solved and the solutions presented in the required forms for analysis.

***Tables summarizing computational solutions.***

| **Supplementary Table S1. Characterization of model T-SR junction Ca^2+^ microdomain** | | | | | | | |
| --- | --- | --- | --- | --- | --- | --- | --- |
| **State of variables:** Ca^2+^ flux into T-SR junction, *Φ*_influx_ and Ca^2+^ flux density into T-SR junction, *J*_influx_ determined from previous experimentally determined maximum rate of [Ca^2+^] increase, d[Ca^2+^]/d*t* and peak cytosolic [Ca^2+^], [Ca^2+^]_max_*,* corresponding to test membrane potential *E* = 0 mV ^1^, using anatomical parameters in Table 1 with the only free parameter in all the computations the value of exit length λ, whose value was then fixed for the remaining computations. | | | | | | | |
| **Name of Variable** | **Definition** | **Value (Physiological units)** | **Dimensions (Physiological units)** | **Value (SI units)** | **Dimensions (SI units)** | **Value (Physiological units)** | **Dimensions (Physiological units)** |
|  | | **6nm mesh** | | | | **3nm mesh** | |
| Ca^2+^ diffusion Parameters | | | | | | | |
| Ca^2+^ diffusion coefficient in **vivo** | *D* | 4 × 10^7^ | nm^2^/s | 4 × 10^-11^ | m^2^/s | 4 × 10^7^ | nm^2^/s |
| Boundary Conditions at 0 mV test voltage: muscle fibre | | | | | | | |
| Maximum rate of [Ca^2+^] increase | d[Ca^2+^]/d*t* | 180 | μmol/(dm^3^s) | 0.18 | mol/(m^3^s) | 180 | μmol/(dm^3^s) |
| Peak cytosolic [Ca^2+^] | [Ca^2+^]_max_ | 3.161 | μmol/dm^3^ | 3.161 × 10^-3^ | mol/m^3^ | 3.161 | μmol/dm^3^ |
| Boundary Conditions at 0 mV test voltage: T-SR junction | | | | | | | |
| Ca^2+^ flux density into T-SR junction | *J_i_*_nflux_ | 3 × 10^-24^ | mol/(nm^2^s) | 3 × 10^-6^ | mol/m^2^/s | 3 × 10^-24^ | mol/(nm^2^s) |
| Ca^2+^ flux into T-SR junction | *Φ*_influx_ *= J*_influx_(π*d^2^/4*) | 1.14 × 10^-19^ | mol/s | 1.14 × 10^-19^ | mol/s | 1.14 × 10^-19^ | mol/s |
| Exit Length | *λ* | 9.2 | nm | 9.2 × 10^-9^ | m | 9.2 | nm |
| Computational parameters | | | | | | | |
| Mesh size | *χ* | 6 | nm | 6 × 10^-9^ | m | 3 | nm |
| Timestep |  | 500 | ns | 5 × 10^-7^ | s | 500 | ns |
| Time resolution |  | 1000 |  | 1000 |  | 1000 |  |
| Time(end) |  | 500 | µs | 5 × 10^-4^ | s | 500 | µs |
| Steady-state solutions, concentrations computed over time | | | | | | | |
| At the centre of the T-SR junction |  | 21.9941 | μmol/dm^3^ | 0.0220 | mol/m^3^ | 21.9603 | μmol/dm^3^ |
| At halfway between centre and edge of T-SR junction |  | 17.2964 | μmol/dm^3^ | 0.0173 | mol/m3 | 17.2597 | μmol/dm^3^ |
| At the edge of the T-SR junction |  | 3.09258 | μmol/dm^3^ | 0.0309 | mol/m^3^ | 3.0892 | μmol/dm^3^ |
| Computed steady-state concentration profiles | | | | | | | |
| [Ca^2+^] at the T-Tubular Membrane | | | | | | | |
| At the centre of the T-SR junction | [Ca^2+^]^T^_centre_ | 21.8694 | μmol/dm^3^ | 0.0219 | mol/m^3^ | 21.8649 | μmol/dm^3^ |
| At halfway between centre and edge of T-SR junction | [Ca^2+^]^T^_50%_ | 17.1447 | μmol/dm^3^ | 0.0171 | mol/m^3^ | 17.1403 | μmol/dm^3^ |
| At the edge of the T-SR junction | [Ca^2+^]^T^_edge_ | 3.0241 | μmol/dm^3^ | 0.0030 | mol/m^3^ | 3.0190 | μmol/dm^3^ |
| Concentration halfway as a proportion of at centre | [Ca^2+^]^T^_50%_ /[Ca^2+^]^T^_centre_ | 0.7840 |  | 0.7840 |  | 0.7839 |  |
| Concentration at edge as a proportion of at centre | [Ca^2+^]^T^_edge_ /[Ca^2+^]^T^_centre_ | 0.1383 |  | 0.1383 |  | 0.1381 |  |
| [Ca^2+^] halfway between the two membranes | | | | | | | |
| At the centre of the T-SR junction | [Ca^2+^]^TSR^_centre_ | 21.9819 | μmol/dm^3^ | 0.0220 | mol/m^3^ | 21.9774 | μmol/dm^3^ |
| At halfway between centre and edge of T-SR junction | [Ca^2+^]^TSR^_50%_ | 17.2572 | μmol/dm^3^ | 0.0173 | mol/m^3^ | 17.2528 | μmol/dm^3^ |
| At the edge of the T-SR junction | [Ca^2+^]^TSR^_edge_ | 3.0926 | μmol/dm^3^ | 0.0031 | mol/m^3^ | 3.0892 | μmol/dm^3^ |
| Concentration halfway as a proportion of at centre | [Ca^2+^]^TSR^_50%_ /[Ca^2+^]^TSR^_centre_ | 0.7851 |  | 0.7851 |  | 0.7850 |  |
| Concentration at edge as a proportion of at centre | [Ca^2+^]^TSR^_edge_ /[Ca^2+^]^TSR^_centre_ | 0.1407 |  | 0.1407 |  | 0.1406 |  |
| [Ca^2+^] at the SR Membrane | | | | | | | |
| At the centre of the T-SR junction | [Ca^2+^]^SR^_centre_ | 22.3194 | μmol/dm^3^ | 0.0223 | mol/m^3^ | 22.3149 | μmol/dm^3^ |
| At halfway between centre and edge of T-SR junction | [Ca^2+^]^SR^_50%_ | 17.5947 | μmol/dm^3^ | 0.0176 | mol/m^3^ | 17.5903 | μmol/dm^3^ |
| At the edge of the T-SR junction | [Ca^2+^]^SR^_edge_ | 3.3591 | μmol/dm^3^ | 0.0034 | mol/m^3^ | 3.3554 | μmol/dm^3^ |
| Concentration halfway as a proportion of at centre | [Ca^2+^]^SR^_50%_ /[Ca^2+^]^SR^_centre_ | 0.7883 |  | 0.7883 |  | 0.7883 |  |
| Concentration at edge as a proportion of at centre | [Ca^2+^]^SR^_edge_ / [Ca^2+^]^SR^_centre_ | 0.1505 |  | 0.1505 |  | 0.1504 |  |
| Relative SR/T membrane [Ca^2+^] | | | | | | | |
| At the centre of the T-SR junction | [Ca^2+^]^SR^_centre_ /[Ca^2+^]^T^_centre_ | 1.020576696 |  | 1.0206 |  | 1.0206 |  |
| At halfway between centre and edge of T-SR junction | [Ca^2+^]^SR^_50%_/ [Ca^2+^]^T^_50%_ | 1.026247178 |  | 1.0262 |  | 1.0263 |  |
| At the edge of the T-SR junction | [Ca^2+^]^SR^_edge_ /[Ca^2+^]^T^_edge_ | 1.11077676 |  | 1.1108 |  | 1.1114 |  |
| Concentration differences between centre and edge | | | | | | | |
| At the T-Tubular Membrane | [Ca^2+^]^ΔT^ | 18.8453 | μmol/dm^3^ | 0.0188 | mol/m^3^ | 18.8460 | μmol/dm^3^ |
| At halfway between the two membranes | [Ca^2+^]^ΔTSR^ | 18.8893 | μmol/dm^3^ | 0.0189 | mol/m^3^ | 18.8883 | μmol/dm^3^ |
| At the SR Membrane | [Ca^2+^]^ΔSR^ | 18.9603 | μmol/dm^3^ | 0.0190 | mol/m^3^ | 18.9596 | μmol/dm^3^ |
| Radial distances for [Ca^2+^] to fall to 50% of centre value | | | | | | | |
| At the T-Tubular Membrane | *X_T_* | 83.6691 | nm | 8.3669× 10^-8^ | m | 83.6606 | nm |
| At halfway between the two membranes | *X_TSR_* | 83.8845 | nm | 8.3885 × 10^-8^ | m | 83.8760 | nm |
| At the SR Membrane | *X_SR_* | 84.5249 | nm | 8.4525 × 10^-8^ | m | 84.5164 | nm |
| Radial distances for [Ca^2+^] to fall to 50% between centre and edge value | | | | | | | |
| At the T-Tubular Membrane | *X’_T_* | 77.6692 | nm | 7.7669 × 10^-8^ | m | 77.6706 | nm |
| At halfway between the two membranes | *X’_TSR_* | 77.7601 | nm | 7.7760 × 10^-8^ | m | 77.7579 | nm |
| At the SR Membrane | *X’_SR_* | 77.9066 | nm | 7.7907 × 10^-8^ | m | 77.9050 | nm |

| **Supplementary Table S2: T-SR junction** **Ca^2+^ microdomain properties at different test voltages** | | | | | | | | |
| --- | --- | --- | --- | --- | --- | --- | --- | --- |
| **State of Variables:** *Values held constant:* Boundary conditions: in vivo Ca^2+^ diffusion coefficient, *D* = 4 × 10^-7^ nm^2^/s; exit length, λ = 9.2 nm, determined as described in Supplementary Table 1. *Computational conditions:* Mesh size = 6 nm; step size = 500 ns; time resolution (number of steps/computation run) = 1000; computational endpoint = 500 μs. *Values varied*: test membrane potentials, *E*, and the corresponding Ca^2+^ influx, *Φ*_influx_ and flux density *J*_influx_ determined from maximum rates of [Ca^2+^] increase, d[Ca^2+^]/d*t*, using anatomical parameters defined in Table 1. | | | | | | | | |
| **Name of Variable (Column)** | **Definition** | **Dimensions (Physiological)** | **Range of test voltages** | | | | | |
| Boundary Conditions over range of varied test voltages: muscle fibre ^1^ | | | | | | | | |
| Test membrane potential | *E* | mV | **-45** | **-40** | **-30** | **-20** | **-10** | **0** |
| Maximum rate of [Ca^2+^] increase | *d*[Ca^2+^]/d*t* | μmol/(dm^3^s) | 3.55 | 18.0 | 90.0 | 120.0 | 170.0 | 180.0 |
| Peak cytosolic Calcium concentration | [Ca^2+^]_max_ | μmol/dm^3^ | 0.135 | 0.511 | 1.711 | 2.427 | 2.858 | 3.161 |
| Boundary Conditions over range of varied test voltages: T-SR junction | | | | | | | | |
| Ca^2+^ flux density into T-SR junction | *J*_influx_ | mol/(nm^2^s) | 5.92 × 10^-26^ | 3.00 × 10^-25^ | 1.50 × 10^-24^ | 2.00 × 10^-24^ | 2.83 × 10^-24^ | 3.00 × 10^-24^ |
| Ca^2+^ flux into T-SR junction | *Φ*_influx_ | mol/s | 2.25 × 10^-21^ | 1.14 × 10^-20^ | 5.70 × 10^-20^ | 7.60 × 10^-20^ | 1.07 × 10^-19^ | 1.14 × 10^-19^ |
| Steady-state solutions, concentrations computed over time | | | | | | | | |
| At the centre of the T-SR junction |  | μmol/dm^3^ | 0.4340 | 2.1994 | 10.9970 | 15.9090 | 20.7477 | 21.9941 |
| At halfway between centre and edge of T-SR junction |  | μmol/dm^3^ | 0.3413 | 1.7296 | 8.6482 | 12.5111 | 16.3163 | 17.2964 |
| At the edge of the T-SR junction |  | μmol/dm^3^ | 0.0610 | 0.3093 | 1.5463 | 2.2370 | 2.9173 | 3.0926 |
| Computed steady-state concentration profiles | | | | | | | | |
| [Ca^2+^] at the T-Tubular Membrane | | | | | | | | |
| At the centre of the T-SR junction | [Ca^2+^]^T^_centre_ | μmol/dm^3^ | 0.4316 | 2.1869 | 10.9347 | 15.8189 | 20.6301 | 21.8694 |
| At halfway between centre and edge of T-SR junction | [Ca^2+^]^T^_50%_ | μmol/dm^3^ | 0.3383 | 1.7145 | 8.5724 | 12.4014 | 16.1732 | 17.1447 |
| At the edge of the T-SR junction | [Ca^2+^]^T^_edge_ | μmol/dm^3^ | 0.0597 | 0.3024 | 1.5121 | 2.1874 | 2.8527 | 3.0241 |
| Concentration halfway as a proportion of at centre | [Ca^2+^]^T^_50%_ / [Ca^2+^]^T^_centre_ |  | 0.7840 | 0.7840 | 0.7840 | 0.7840 | 0.7840 | 0.7840 |
| Concentration at edge as a proportion of at centre | [Ca^2+^]^T^_edge_ / [Ca^2+^]^T^_centre_ |  | 0.1383 | 0.1383 | 0.1383 | 0.1383 | 0.1383 | 0.1383 |
| [Ca^2+^] halfway between the two membranes | | | | | | | | |
| At the centre of the T-SR junction | [Ca^2+^]^TSR^_centre_ | μmol/dm^3^ | 0.4338 | 2.1982 | 10.9909 | 15.9002 | 20.7363 | 21.9819 |
| At halfway between centre and edge of T-SR junction | [Ca^2+^]^TSR^_50%_ | μmol/dm^3^ | 0.3405 | 1.7257 | 8.6286 | 12.4827 | 16.2793 | 17.2572 |
| At the edge of the T-SR junction | [Ca^2+^]^TSR^_edge_ | μmol/dm^3^ | 0.0610 | 0.3093 | 1.5463 | 2.2370 | 2.9173 | 3.0926 |
| Concentration halfway as a proportion of at centre | [Ca^2+^]^TSR^_50%_ /[Ca^2+^]^TSR^_centre_ |  | 0.7851 | 0.7851 | 0.7851 | 0.7851 | 0.7851 | 0.7851 |
| Concentration at edge as a proportion of at centre | [Ca^2+^]^TSR^_edge_ /[Ca^2+^]^TSR^_centre_ |  | 0.1407 | 0.1407 | 0.1407 | 0.1407 | 0.1407 | 0.1407 |
| [Ca^2+^] at the SR Membrane | | | | | | | | |
| At the centre of the T-SR junction | [Ca^2+^]^SR^_centre_ | μmol/dm^3^ | 0.4404 | 2.2319 | 11.1597 | 16.1444 | 21.0546 | 22.3194 |
| At halfway between centre and edge of T-SR junction | [Ca^2+^]^SR^_50%_ | μmol/dm^3^ | 0.3472 | 1.7595 | 8.7974 | 12.7269 | 16.5977 | 17.5947 |
| At the edge of the T-SR junction | [Ca^2+^]^SR^_edge_ | μmol/dm^3^ | 0.0663 | 0.3359 | 1.6795 | 2.4297 | 3.1687 | 3.3591 |
| Concentration halfway as a proportion of at centre | [Ca^2+^]^SR^_50%_ /[Ca^2+^]^SR^_centre_ |  | 0.7883 | 0.7883 | 0.7883 | 0.7883 | 0.7883 | 0.7883 |
| Concentration at edge as a proportion of at centre | [Ca^2+^]^SR^_edge_ /[Ca^2+^]^SR^_centre_ |  | 0.1505 | 0.1505 | 0.1505 | 0.1505 | 0.1505 | 0.1505 |
| Relative SR/T membrane [Ca^2+^] | | | | | | | | |
| At the centre of the T-SR junction | [Ca^2+^]^SR^_centre_ /[Ca^2+^]^T^_centre_ |  | 1.0206 | 1.0206 | 1.0206 | 1.0206 | 1.0206 | 1.0206 |
| At halfway between centre and edge of T-SR junction | [Ca^2+^]^SR^_50%_/ [Ca^2+^]^T^_50%_ |  | 1.0262 | 1.0262 | 1.0262 | 1.0262 | 1.0262 | 1.0262 |
| At the edge of the T-SR junction | [Ca^2+^]^SR^_edge_ /[Ca^2+^]^T^_edge_ |  | 1.1108 | 1.1108 | 1.1108 | 1.1108 | 1.1108 | 1.1108 |
| Concentration differences between centre and edge | | | | | | | | |
| At the T-Tubular Membrane | [Ca^2+^]^ΔT^ | μmol/dm^3^ | 0.3719 | 1.8845 | 9.4227 | 13.6314 | 17.7774 | 18.8453 |
| At halfway between the two membranes | [Ca^2+^]^ΔTSR^ | μmol/dm^3^ | 0.3727 | 1.8889 | 9.4447 | 13.6633 | 17.8189 | 18.8893 |
| At the SR Membrane | [Ca^2+^]^ΔSR^ | μmol/dm^3^ | 0.3742 | 1.896 | 9.4802 | 13.7146 | 17.8859 | 18.9603 |
| Radial distances for [Ca^2+^] to fall to 50% of centre value | | | | | | | | |
| At the T-Tubular membrane | *X_T_* | nm | 83.6691 | 83.6691 | 83.6691 | 83.6691 | 83.6691 | 83.6691 |
| At halfway between the two membranes | *X_TSR_* | nm | 83.8845 | 83.8845 | 84.5249 | 83.8845 | 83.8845 | 83.8845 |
| At the SR Membrane | *X_SR_* | nm | 84.5249 | 84.5249 | 84.5249 | 84.5249 | 84.5249 | 84.5249 |
| Radial distances for [Ca^2+^] to fall to 50% between centre and edge value | | | | | | | | |
| At the T-Tubular membrane | *X’_T_* | nm | 77.6692 | 77.6692 | 77.6692 | 77.6692 | 77.6692 | 77.6692 |
| At halfway between the two membranes | *X’_TSR_* | nm | 77.7601 | 77.7601 | 77.7601 | 77.7601 | 77.7601 | 77.7601 |
| At the SR Membrane | *X’_SR_* | nm | 77.9066 | 77.9066 | 77.9066 | 77.9066 | 77.9066 | 77.9066 |

| **Supplementary Table S3: T-SR junction Ca^2+^ microdomains at sub-threshold Ca^2+^ flux densities** | | | | | |
| --- | --- | --- | --- | --- | --- |
| **State of Variables:** *Values held constant:* Boundary conditions: in vivo Ca^2+^ diffusion coefficient, *D* = 4 × 10^-7^ nm^2^/s; exit length, λ = 9.2 nm, determined as described in Table 2. Anatomical parameters as defined in Table 1. *Computational conditions:* Mesh size = 6 nm; step size = 500 ns; time resolution (number of steps/computational run) = 1000; computational endpoint = 500 μs. *Values varied*: flux density *J*_influx_ and corresponding Ca^2+^ influx, *Φ*_influx_. | | | | | |
| **Name of Variable (Column)** | **Definition** | **Dimensions (Physiological)** | **Calcium flux densities into the T-SR space explored** | | |
| Boundary conditions over a range of varied test voltages: T-SR Junction | | | | | |
| Ca^2+^ flux density into T-SR junction | *J_i_*_nflux_ | mol/(nm^2^s) | 5.92 × 10^-26^ | 5.92 × 10^-27^ | 5.92 × 10^-28^ |
| Ca^2+^ flux into T-SR junction | *Φ_i_*_nflux_ | mol/s | 2.25 × 10^-21^ | 2.25 × 10^-22^ | 2.25 × 10^-23^ |
| Steady-state solutions, concentrations computed over time | | | | | |
| At the centre of the T-SR junction |  | μmol/dm^3^ | 0.434 | 0.0434 | 0.0043 |
| At halfway between centre and edge of T-SR junction |  | μmol/dm^3^ | 0.3413 | 0.0341 | 0.0034 |
| At the edge of the T-SR junction |  | μmol/dm^3^ | 0.061 | 0.0061 | 0.0006 |
| Computed steady-state concentration profiles | | | | | |
| [Ca^2+^] at the T-Tubular Membrane | | | | | |
| At the centre of the T-SR junction | [Ca^2+^]^T^_centre_ | μmol/dm^3^ | 0.4316 | 0.0432 | 0.0043 |
| At halfway between centre and edge of T-SR junction | [Ca^2+^]^T^_50%_ | μmol/dm^3^ | 0.3383 | 0.0338 | 0.0034 |
| At the edge of the T-SR junction | [Ca^2+^]^T^_edge_ | μmol/dm^3^ | 0.0597 | 0.006 | 0.0006 |
| Concentration halfway as a proportion of at centre | [Ca^2+^]^T^_50%_ / [Ca^2+^]^T^_centre_ |  | 0.784 | 0.784 | 0.784 |
| Concentration at edge as a proportion of at centre | [Ca^2+^]^T^_edge_ / [Ca^2+^]^T^_centre_ |  | 0.1383 | 0.1383 | 0.1383 |
| [Ca^2+^] halfway between the two membranes | | | | | |
| At the centre of the T-SR junction | [Ca^2+^]^TSR^_centre_ | μmol/dm^3^ | 0.4338 | 0.0434 | 0.0043 |
| At halfway between centre and edge of T-SR junction | [Ca^2+^]^TSR^_50%_ | μmol/dm^3^ | 0.3405 | 0.0341 | 0.0034 |
| At the edge of the T-SR junction | [Ca^2+^]^TSR^_edge_ | μmol/dm^3^ | 0.061 | 0.0061 | 0.0006 |
| Concentration halfway as a proportion of at centre | [Ca^2+^]^TSR^_50%_ /[Ca^2+^]^TSR^_centre_ |  | 0.7851 | 0.7851 | 0.7851 |
| Concentration at edge as a proportion of at centre | [Ca^2+^]^TSR^_edge_ / [Ca^2+^]^TSR^_centre_ |  | 0.1407 | 0.1407 | 0.1407 |
| [Ca^2+^] at the SR Membrane | | | | | |
| At the centre of the T-SR junction | [Ca^2+^]^SR^_centre_ | μmol/dm^3^ | 0.4404 | 0.044 | 0.0044 |
| At halfway between centre and edge of T-SR junction | [Ca^2+^]^SR^_50%_ | μmol/dm^3^ | 0.3472 | 0.0347 | 0.0035 |
| At the edge of the T-SR junction | [Ca^2+^]^SR^_edge_ | μmol/dm^3^ | 0.0663 | 0.0066 | 0.0007 |
| Concentration halfway as a proportion of at centre | [Ca^2+^]^SR^_50%_ / [Ca^2+^]^SR^_centre_ |  | 0.7883 | 0.7883 | 0.7883 |
| Concentration at edge as a proportion of at centre | [Ca^2+^]^SR^_edge_ / [Ca^2+^]^SR^_centre_ |  | 0.1505 | 0.1505 | 0.1505 |
| Relative SR/T membrane [Ca^2+^] | | | | | |
| At the centre of the T-SR junction | [Ca^2+^]^SR^_centre_ /[Ca^2+^]^T^_centre_ |  | 1.0206 | 1.0206 | 1.0206 |
| At halfway between centre and edge of T-SR junction | [Ca^2+^]^SR^_50%_/ [Ca^2+^]^T^_50%_ |  | 1.0262 | 1.0262 | 1.0262 |
| At the edge of the T-SR junction | [Ca^2+^]^SR^_edge_ /[Ca^2+^]^T^_edge_ |  | 1.1108 | 1.1108 | 1.1108 |
| Concentration differences between centre and edge | | | | | |
| At the T-Tubular Membrane | [Ca^2+^]^ΔT^ | μmol/dm^3^ | 0.3719 | 0.0372 | 0.0037 |
| At halfway between the two membranes | [Ca^2+^]^ΔTSR^ | μmol/dm^3^ | 0.3727 | 0.0373 | 0.0037 |
| At the SR Membrane | [Ca^2+^]^ΔSR^ | μmol/dm^3^ | 0.3742 | 0.0374 | 0.0037 |
| Radial distances for [Ca^2+^] to fall to 50% of centre value | | | | | |
| At the T-Tubular membrane | *X_T_* | nm | 83.6691 | 83.6691 | 83.6691 |
| At halfway between the two membranes | *X_TSR_* | nm | 83.8845 | 83.8845 | 83.8845 |
| At the SR Membrane | *X_SR_* | nm | 84.5249 | 84.5249 | 84.5249 |
| Radial distances for [Ca^2+^] to fall to 50% between centre and edge value | | | | | |
| At the T-Tubular membrane | *X’_T_* | nm | 77.6692 | 77.6692 | 77.6692 |
| At halfway between the two membranes | *X’_TSR_* | nm | 77.7601 | 77.7601 | 77.7601 |
| At the SR Membrane | *X’_SR_* | nm | 77.9066 | 77.9066 | 77.9066 |


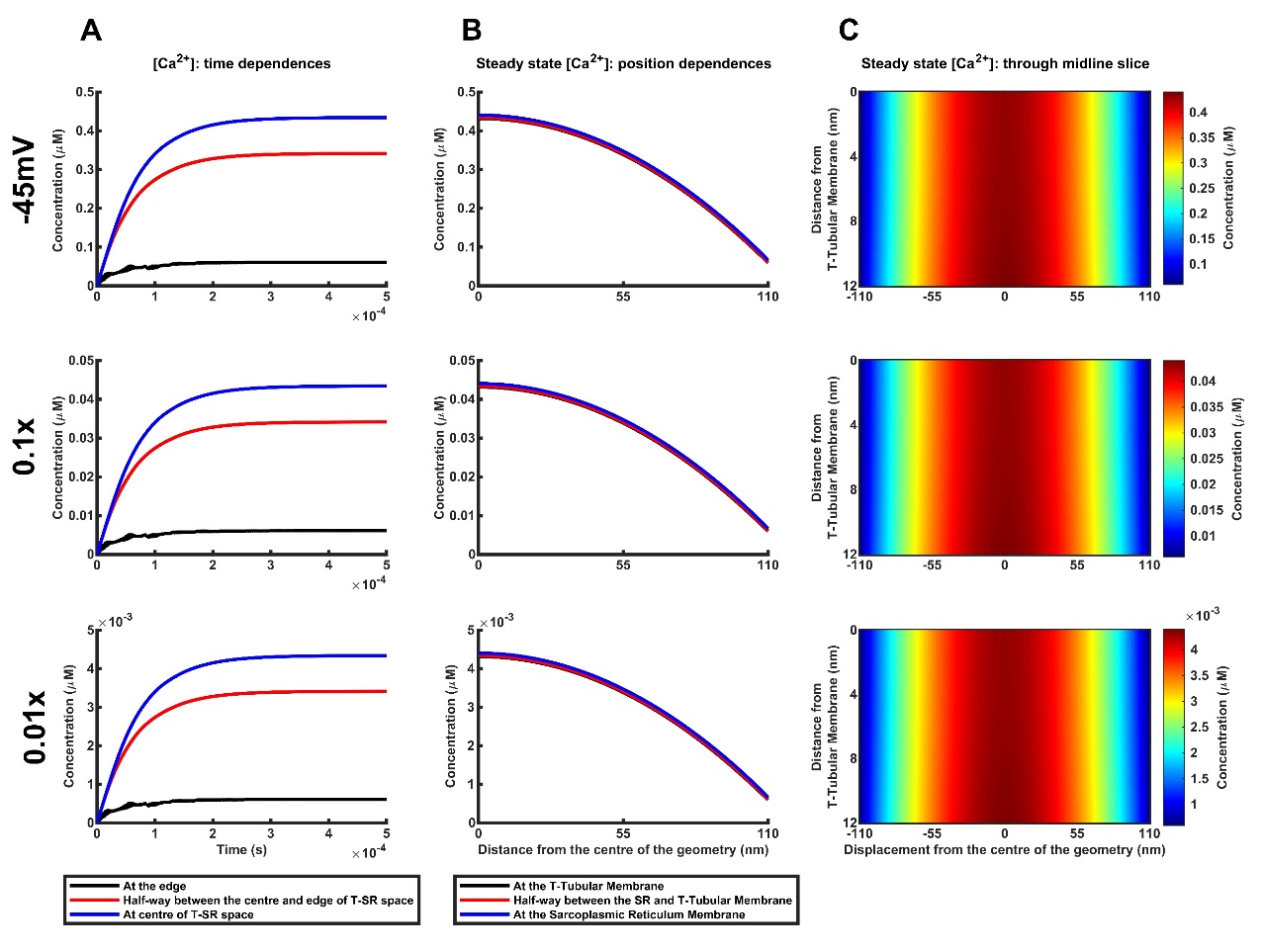


**Supplementary Figure S2. *Properties of Ca^2+^ microdomains at reduced T-SR junction Ca^2+^ influx densities.***

Explorations for and characterizations of [Ca^2+^] microdomains at subthreshold Ca^2+^ influx densities, $J_{\mathrm{influx}}$. (A) Variations in [Ca^2+^] with time following onset of imposed $J_{\mathrm{influx}}$. (B) Dependence of the resulting steady state [Ca^2+^] upon radial distance from the centre of the T-SR junction. (C) Corresponding mapping of [Ca^2+^] across a midline axial slice; values of [Ca^2+^] scaled linearly with $\Phi_{\mathrm{influx}}$ to illustrate features of microdomain characteristics and radial concentration gradients. Successive rows (top to bottom) show results from values of $J_{\mathrm{influx}}$computed for previously reported threshold levels (at test voltage – 45 mV), and $J_{\mathrm{influx}}$values two successive orders of magnitude lower.

| **Supplementary Table S4: T-SR junction Ca^2+^ microdomains produced with varying diffusion coefficients.** | | | | | | | |
| --- | --- | --- | --- | --- | --- | --- | --- |
| **State of Variables:** *Values held constant:* Boundary conditions as determined in Table 2: Test membrane potential *E* = 0 mV; Maximum rate of [Ca^2+^] increase, d[Ca^2+^]/dt = 180 µM/s (Kovacs et al., 1983). Ca^2+^ flux density into T-SR junction, *J*_influx_ = 3.00 × 10^-24^ mol/(nm^2^s); Ca^2+^ influx into T-SR junction, *Φ*_influx_ = *J*_influx_(π*d*^2^/4) = 1.14 × 10^-19^ mol/s. Exit length, λ = 9.2 nm. Anatomical parameters including constant T-SR distance*, w*, and T-SR diameter *d*, defined in Table 1. Computational parameters: Mesh size = 6 nm, time resolution (number of steps/computational run) = 10000*. Values varied:* Ca^2+^ diffusion coefficient, *D*, computational timestep and end time. | | | | | | | |
| **Name of Variable (Column)** | **Definition** | **Dimensions (Physiological)** | **Range of Ca^2+^ diffusion coefficient, D.** | | | | |
| Calcium diffusion parameters | | | | | | | |
| Ca^2+^ diffusion coefficient | *D* | nm^2^/s | 1 × 10^9^ | 5.2 × 10^8^ | 4.0 × 10^7^ | 4.0 × 10^6^ | 4.0 × 10^5^ |
| Computational parameters | | | | | | | |
| Mesh size | *χ* | nm | 6 | 6 | 6 | 6 | 6 |
| Timestep |  | ns | 5 | 10 | 100 | 1 × 10^3^ | 1 × 10^4^ |
| Time resolution |  |  | 10000 | 10000 | 10000 | 10000 | 10000 |
| Time(end) |  | μs | 50 | 100 | 1 × 10^3^ | 1 × 10^4^ | 1 × 10^5^ |
| Steady-state solutions, concentrations computed over time | | | | | | | |
| At the centre of the T-SR junction |  | μmol/dm^3^ | 0.88 | 1.6924 | 22.0008 | 220 | 2200.08 |
| At halfway between centre and edge of T-SR junction |  | μmol/dm^3^ | 0.6920 | 1.3309 | 17.3012 | 173.012 | 1730.12 |
| At the edge of the T-SR junction |  | μmol/dm^3^ | 0.1237 | 0.2379 | 3.0933 | 30.9328 | 309.328 |
| Computed steady-state concentration profiles | | | | | | | |
| [Ca^2+^] at the T-Tubular Membrane | | | | | | | |
| At the centre of the T-SR junction | [Ca^2+^]^T^_centre_ | μmol/dm^3^ | 0.8750 | 1.6828 | 21.8761 | 218.761 | 2187.61 |
| At halfway between centre and edge of T-SR junction | [Ca^2+^]^T^_50%_ | μmol/dm^3^ | 0.6860 | 1.3192 | 17.1495 | 171.495 | 1714.95 |
| At the edge of the T-SR junction | [Ca^2+^]^T^_edge_ | μmol/dm^3^ | 0.1210 | 0.2327 | 3.0248 | 30.248 | 302.48 |
| Concentration halfway as a proportion of at centre | [Ca^2+^]^T^_50%_ /[Ca^2+^]^T^_centre_ |  | 0.7839 | 0.7839 | 0.7839 | 0.7839 | 0.7839 |
| Concentration at edge as a proportion of at centre | [Ca^2+^]^T^_edge_ / [Ca^2+^]^T^_centre_ |  | 0.1383 | 0.1383 | 0.1383 | 0.1383 | 0.1383 |
| [Ca^2+^] halfway between the two membranes | | | | | | | |
| At the centre of the T-SR junction | [Ca^2+^]^TSR^_centre_ | μmol/dm^3^ | 0.8795 | 1.6914 | 21.9886 | 219.886 | 2198.86 |
| At halfway between centre and edge of T-SR junction | [Ca^2+^]^TSR^_50%_ | μmol/dm^3^ | 0.6905 | 1.3279 | 17.262 | 172.62 | 1726.2 |
| At the edge of the T-SR junction | [Ca^2+^]^TSR^_edge_ | μmol/dm^3^ | 0.1237 | 0.2379 | 3.0933 | 30.9328 | 309.328 |
| Concentration halfway as a proportion of at centre | [Ca^2+^]^TSR^_50%_ /[Ca^2+^]^TSR^_centre_ |  | 0.7850 | 0.7850 | 0.7850 | 0.7850 | 0.7850 |
| Concentration at edge as a proportion of at centre | [Ca^2+^]^TSR^_edge_ /[Ca^2+^]^TSR^_centre_ |  | 0.1407 | 0.1407 | 0.1407 | 0.1407 | 0.1407 |
| [Ca^2+^] at the SR Membrane | | | | | | | |
| At the centre of the T-SR junction | [Ca^2+^]^SR^_centre_ | μmol/dm^3^ | 0.8930 | 1.7174 | 22.3261 | 223.261 | 2232.61 |
| At halfway between centre and edge of T-SR junction | [Ca^2+^]^SR^_50%_ | μmol/dm^3^ | 0.7040 | 1.3538 | 17.5995 | 175.995 | 1759.95 |
| At the edge of the T-SR junction | [Ca^2+^]^SR^_edge_ | μmol/dm^3^ | 0.1344 | 0.2584 | 3.3598 | 33.5976 | 335.976 |
| Concentration halfway as a proportion of at centre | [Ca^2+^]^SR^_50%_ /[Ca^2+^]^SR^_centre_ |  | 0.7883 | 0.7883 | 0.7883 | 0.7883 | 0.7883 |
| Concentration at edge as a proportion of at centre | [Ca^2+^]^SR^_edge_ /[Ca^2+^]^SR^_centre_ |  | 0.1505 | 0.1505 | 0.1505 | 0.1505 | 0.1505 |
| Relative SR/T membrane [Ca^2+^] | | | | | | | |
| At the centre of the T-SR junction | [Ca^2+^]^SR^_centre_ /[Ca^2+^]^T^_centre_ |  | 1.0206 | 1.0206 | 1.0206 | 1.0206 | 1.0206 |
| At halfway between centre and edge of T-SR junction | [Ca^2+^]^SR^_50%_/[Ca^2+^]^T^_50%_ |  | 1.0262 | 1.0262 | 1.0262 | 1.0262 | 1.0262 |
| At the edge of the T-SR junction | [Ca^2+^]^SR^_edge_ /[Ca^2+^]^T^_edge_ |  | 1.1107 | 1.1107 | 1.1107 | 1.1107 | 1.1107 |
| Concentration differences between centre and edge | | | | | | | |
| At the T-Tubular Membrane | [Ca^2+^]^ΔT^ | μmol/dm^3^ | 0.7541 | 1.4501 | 18.8513 | 188.513 | 1885.13 |
| At halfway between the two membranes | [Ca^2+^]^ΔTSR^ | μmol/dm^3^ | 0.7558 | 1.4535 | 18.8954 | 188.954 | 1889.54 |
| At the SR Membrane | [Ca^2+^]^ΔSR^ | μmol/dm^3^ | 0.7587 | 1.459 | 18.9664 | 189.664 | 1896.64 |
| Radial distances for [Ca^2+^] to fall to 50% of centre value | | | | | | | |
| At the T-Tubular membrane | *X_T_* | nm | 83.6667 | 83.6667 | 83.6667 | 83.6667 | 83.6667 |
| At halfway between the two membranes | *X_TSR_* | nm | 83.882 | 83.8820 | 83.8820 | 83.8820 | 83.8820 |
| At the SR Membrane | *X_SR_* | nm | 77.667 | 84.5222 | 84.5222 | 84.5222 | 84.5222 |
| Radial distances for [Ca^2+^] to fall to 50% between centre and edge value | | | | | | | |
| At the T-Tubular membrane | *X’_T_* | nm | 77.6670 | 77.6670 | 77.6670 | 77.6670 | 77.6670 |
| At halfway between the two membranes | *X’_TSR_* | nm | 77.7579 | 77.7578 | 77.7578 | 77.7578 | 77.7578 |
| At the SR Membrane | *X’_SR_* | nm | 77.9044 | 77.9044 | 77.9044 | 77.9044 | 77.9044 |

*References to Ca^2+^ diffusion coefficient values (in nm^2^/s) used:* Free diffusion coefficient infinite dilution: 1 × 10^9^ (ref.^2^); Free diffusion coefficient at axoplasmic concentrations: 5.2 × 10^8^ (ref. ^3^); Muscle cytoplasm: 4.0 × 10^7^ (ref. ^4–6^; Lower values explored: 4.0 × 10^6^ and 4.0 × 10^5^*. Previous literature values:* Unbuffered media, infinite dilution: 1.0 × 10^9^ (ref.^2^); Aqueous CaCl2 solution: 7.78 × 10^8^ (ref. ^7^); Nerve cell body: 8.3 × 10^7^ (ref. ^8^); Electrolyte solution: 5.2 × 10^8^ (ref. ^8^); ^45^Ca measurements: 5.3 × 10^8^ (ref. ^3^); Axoplasm, organelles intact: 1.0 × 10^7^ (ref. ^9^); Axoplasm, ATP depleted: 5.0 × 10^7^ to 2.0 × 10^8^ (ref. ^9^); Amphibian muscle: 3.0 × 10^8^ (ref. ^4^); 1.4 × 10^7^ (ref. ^6^); 3.2 × 10^8^ (ref. ^5^); Mammalian cardiac muscle: 3.0 × 10^8^ (ref. ^10^); 1.0 × 10^7^ to 2.0 × 10^7^ (ref. ^11^).

| **Supplementary Table S5: Ca^2+^ microdomains at varied T-SR junction axial distances** | | | | | | |
| --- | --- | --- | --- | --- | --- | --- |
| **State of Variables:** *Values held constant:* Boundary conditions as determined in Table 2: Test membrane potential *E* = 0 mV; Maximum rate of [Ca^2+^] increase, d[Ca^2+^]/d*t* = 180 µM/s; (Kovacs et al., 1983). Ca^2+^ flux density into T-SR junction, *J*_influx_ = 3.00 × 10^-24^ mol/(nm^2^ s); Ca^2+^ flux into T-SR junction, *Φ*_influx_ = *J*_influx_ (π*d*^2^/4) = 1.14 × 10^-19^ mol/s. Ca^2+^ diffusion coefficient *D* = 4 × 10^7^ nm^2^/s; Exit length, λ = 9.2 nm. Anatomical parameters including constant T-SR diameter *d*, defined in Table 1. *Values varied:* T-SR distance, *w,* mesh size, time resolution, timestep. | | | | | | |
| ***Name of Variable (Column)*** | **Definition** | **Dimensions (Physiological)** | **T-SR distances explored** | | | |
| T-SR distances explored | | | | | | |
| T-SR distance | *w* | nm | 6 | 12 | 20 | 30 |
| Computational parameters | | | | | | |
| Mesh size | *χ* | nm | 5 | 3 | 5 | 7.5 |
| Timestep |  | ns | 50 | 500 | 500 | 500 |
| Time resolution |  |  | 10000 | 1000 | 1000 | 1000 |
| Time(end) |  | μs | 500 | 500 | 500 | 500 |
| Steady-state solutions, concentrations computed over time | | | | | | |
| At the centre of the T-SR junction |  | μmol/dm^3^ | 44.0208 | 21.9603 | 13.1481 | 8.6983 |
| At halfway between centre and edge of T-SR junction |  | μmol/dm^3^ | 34.105 | 17.2597 | 10.3036 | 6.7946 |
| At the edge of the T-SR junction |  | μmol/dm^3^ | 6.2294 | 3.0892 | 1.8256 | 1.1885 |
| Computed steady-state concentration profiles | | | | | | |
| [Ca^2+^] at the T-Tubular Membrane | | | | | | |
| At the centre of the T-SR junction | [Ca^2+^]^T^_centre_ | μmol/dm^3^ | 43.9646 | 21.8649 | 12.9589 | 8.4284 |
| At halfway between centre and edge of T-SR junction | [Ca^2+^]^T^_50%_ | μmol/dm^3^ | 34.5153 | 17.1403 | 10.1238 | 6.5395 |
| At the edge of the T-SR junction | [Ca^2+^]^T^_edge_ | μmol/dm^3^ | 6.19 | 3.019 | 1.7351 | 1.0811 |
| Concentration halfway as a proportion of at centre | [Ca^2+^]^T^_50%_ / [Ca^2+^]^T^_centre_ |  | 0.7851 | 0.7839 | 0.7812 | 0.7759 |
| Concentration at edge as a proportion of at centre | [Ca^2+^]^T^_edge_ / [Ca^2+^]^T^_centre_ |  | 0.1408 | 0.1381 | 0.1339 | 0.1283 |
| [Ca^2+^] halfway between the two membranes | | | | | | |
| At the centre of the T-SR junction | [Ca^2+^]^TSR^_centre_ | μmol/dm^3^ | 44.0209 | 21.9774 | 13.1464 | 8.7096 |
| At halfway between centre and edge of T-SR junction | [Ca^2+^]^TSR^_50%_ | μmol/dm^3^ | 34.5715 | 17.2528 | 10.3113 | 6.8196 |
| At the edge of the T-SR junction | [Ca^2+^]^TSR^_edge_ | μmol/dm^3^ | 6.2294 | 3.0892 | 1.8256 | 1.1885 |
| Concentration halfway as a proportion of at centre | [Ca^2+^]^TSR^_50%_ / [Ca^2+^]^TSR^_centre_ |  | 0.7853 | 0.785 | 0.7843 | 0.783 |
| Concentration at edge as a proportion of at centre | [Ca^2+^]^TSR^_edge_ / [Ca^2+^]^TSR^_centre_ |  | 0.1415 | 0.1406 | 0.1389 | 0.1365 |
| [Ca^2+^] at the SR Membrane | | | | | | |
| At the centre of the T-SR junction | [Ca^2+^]^SR^_centre_ | μmol/dm^3^ | 44.1896 | 22.3149 | 13.7089 | 9.5533 |
| At halfway between centre and edge of T-SR junction | [Ca^2+^]^SR^_50%_ | μmol/dm^3^ | 34.7403 | 17.5903 | 10.8738 | 7.6623 |
| At the edge of the T-SR junction | [Ca^2+^]^SR^_edge_ | μmol/dm^3^ | 6.3819 | 3.3554 | 2.2125 | 1.6917 |
| Concentration halfway as a proportion of at centre | [Ca^2+^]^SR^_50%_ / [Ca^2+^]^SR^_centre_ |  | 0.7862 | 0.7883 | 0.7932 | 0.8021 |
| Concentration at edge as a proportion of at centre | [Ca^2+^]^SR^_edge_ / [Ca^2+^]^SR^_centre_ |  | 0.1444 | 0.1504 | 0.1614 | 0.1771 |
| Relative SR/T membrane [Ca^2+^] | | | | | | |
| At the centre of the T-SR junction | [Ca^2+^]^SR^_centre_ /[Ca^2+^]^T^_centre_ |  | 1.0051 | 1.0206 | 1.0579 | 1.1335 |
| At halfway between centre and edge of T-SR junction | [Ca^2+^]^SR^_50%_/ [Ca^2+^]^T^_50%_ |  | 1.0065 | 1.0263 | 1.0741 | 1.1717 |
| At the edge of the T-SR junction | [Ca^2+^]^SR^_edge_ /[Ca^2+^]^T^_edge_ |  | 1.031 | 1.1114 | 1.2751 | 1.5648 |
| Concentration differences between centre and edge | | | | | | |
| At the T-Tubular Membrane | [Ca^2+^]^ΔT^ | μmol/dm^3^ | 37.7746 | 18.8460 | 11.2238 | 7.34724 |
| At halfway between the two membranes | [Ca^2+^]^ΔTSR^ | μmol/dm^3^ | 37.7914 | 18.8883 | 11.3208 | 7.52116 |
| At the SR Membrane | [Ca^2+^]^ΔSR^ | μmol/dm^3^ | 37.8077 | 18.9596 | 11.4964 | 7.8616 |
| Radial distances for [Ca^2+^] to fall to 50% of centre value | | | | | | |
| At the T-Tubular membrane | *X_T_* | nm | 83.8848 | 83.6606 | 83.1585 | 82.2741 |
| At halfway between the two membranes | *X_TSR_* | nm | 83.9388 | 83.876 | 83.7436 | 83.4879 |
| At the SR Membrane | *X_SR_* | nm | 84.0993 | 84.5164 | 85.4968 | 87.2079 |
| Radial distances to fall to 50% as a fraction of total TSR Radius | | | | | | |
| At the T-Tubular membrane | *2X_T_ /d* |  | 0.7626 | 0.7606 | 0.756 | 0.7479 |
| At halfway between the two membranes, | *2X_TSR_ /d* |  | 0.7631 | 0.7625 | 0.7613 | 0.759 |
| At the SR Membrane, | *2X_SR_ /d* |  | 0.7645 | 0.7683 | 0.7772 | 0.7928 |
| Radial distances for [Ca^2+^] to fall to 50% between centre and edge value | | | | | | |
| At the T-Tubular membrane | *X’_T_* | nm | 77.7555 | 77.6706 | 77.384 | 76.7704 |
| At halfway between the two membranes | *X’_TSR_* | nm | 77.7729 | 77.7579 | 77.7118 | 77.5811 |
| At the SR Membrane | *X’_SR_* | nm | 77.7897 | 77.905 | 78.305 | 79.2044 |

Previously reported T-SR distances (nm): Amphibian muscle at rest; in isotonic solution: 12.0 (ref. ^12^); 16.59 ± 0.127 (ref. ^13^). In hypotonic Ringer solution: 29.6 ± 0.204 (ref. ^13^). In hypertonic extracellular solutions: 6.60 ± 0.41 (ref. ^14^); Fatigue; low frequency intermittent stimulation 20.15 ± 0.56 (ref. ^15^).

| **Supplementary Table S6: Ca^2+^ microdomains at varied T-SR junction diameters** | | | | | | | |
| --- | --- | --- | --- | --- | --- | --- | --- |
| **State of Variables:** *Values held constant:* Boundary conditions as determined in Table 2: Test membrane potential *E* = 0 mV; Maximum rate of [Ca^2+^] increase, d[Ca^2+^]/dt = 180 µM/s; (Kovacs et al., 1983). Ca^2+^ flux density into T-SR junction, *J*_influx_ = 3.00 × 10^-24^ mol/(nm^2^ s); Ca^2+^ flux into T-SR junction,  *Φ*_influx_ = *J*_influx_(πd^2^/4) = 1.14 × 10^-19^ mol/s; Exit length, λ = 9.2 nm; Ca^2+^ diffusion coefficient *D* = 4 × 10^7^ nm^2^/s; time resolution (number of steps/computation run) = 1000. Anatomical parameters including constant T-SR distance*, w* = 12 nm, defined in Table 1. *Values varied:* T-SR diameter, d, mesh size, timestep, time (end). | | | | | | | |
| **Name of Variable (Column)** | **Definition** | **Dimensions (Physiological)** | **T-SR diameters explored** | | | | |
| T-SR diameters | | | | | | | |
| T-SR diameter | *d* | nm | **220** | **160** | **100** | **60** | **40** |
| Boundary Conditions over range of varied T-SR diameters | | | | | | | |
| Ca^2+^ flux density into T-SR junction | *J_influx_* | mol/(nm^2^s) | 3 × 10^-24^ | 3 × 10^-24^ | 3 × 10^-24^ | 3 × 10^-24^ | 3 × 10^-24^ |
| Ca^2+^ flux into T-SR junction | *Φ_influx_* | mol/s | 1.14 × 10^-19^ | 6.0319 × 10^-20^ | 2.3562 × 10^-20^ | 8.4823 × 10^-21^ | 3.7699 × 10^-21^ |
| Computational parameters | | | | | | | |
| Mesh size | *χ* | nm | 6 | 6 | 6 | 6 | 3 |
| Timestep |  | ns | 100 | 50 | 50 | 50 | 50 |
| Time resolution |  |  | 1000 | 1000 | 1000 | 1000 | 1000 |
| Time(end) |  | μs | 100 | 500 | 500 | 500 | 500 |
| Steady-state solutions, concentrations computed over time | | | | | | | |
| At the centre of the T-SR junction |  | μmol/dm^3^ | 22.0008 | 12.214 | 5.3158 | 2.2169 | 1.1542 |
| At halfway between centre and edge of T-SR junction |  | μmol/dm^3^ | 17.3012 | 9.8645 | 4.4244 | 1.8486 | 1.0126 |
| At the edge of the T-SR junction |  | μmol/dm^3^ | 3.0933 | 2.2466 | 1.3909 | 0.82 | 0.5409 |
| Computed steady-state concentration profiles | | | | | | | |
| [Ca^2+^] at the T-Tubular Membrane | | | | | | | |
| At the centre of the T-SR junction | [Ca^2+^]^T^_centre_ | μmol/dm^3^ | 21.8761 | 12.1239 | 5.1773 | 2.105 | 1.0472 |
| At halfway between centre and edge of T-SR junction | [Ca^2+^]^T^_50%_ | μmol/dm^3^ | 17.1495 | 9.624 | 4.2008 | 1.7549 | 0.8954 |
| At the edge of the T-SR junction | [Ca^2+^]^T^_edge_ | μmol/dm^3^ | 3.0248 | 2.1781 | 1.3239 | 0.7523 | 0.4726 |
| Concentration halfway as a proportion of at centre | [Ca^2+^]^T^_50%_ /[Ca^2+^]^T^_centre_ |  | 0.7839 | 0.7938 | 0.8114 | 0.8336 | 0.855 |
| Concentration at edge as a proportion of at centre | [Ca^2+^]^T^_edge_ /[Ca^2+^]^T^_centre_ |  | 0.1383 | 0.1797 | 0.2557 | 0.3574 | 0.4513 |
| [Ca^2+^] halfway between the two membranes | | | | | | | |
| At the centre of the T-SR junction | [Ca^2+^]^TSR^_centre_ | μmol/dm^3^ | 21.9886 | 12.2364 | 5.2898 | 2.2174 | 1.1579 |
| At halfway between centre and edge of T-SR junction | [Ca^2+^]^TSR^_50%_ | μmol/dm^3^ | 17.262 | 9.7365 | 4.3132 | 1.8658 | 1.0018 |
| At the edge of the T-SR junction | [Ca^2+^]^TSR^_edge_ | μmol/dm^3^ | 3.0933 | 2.2466 | 1.3909 | 0.82 | 0.5409 |
| Concentration halfway as a proportion of at centre | [Ca^2+^]^TSR^_50%_/[Ca^2+^]^TSR^_centre_ |  | 0.785 | 0.7957 | 0.8154 | 0.8414 | 0.8651 |
| Concentration at edge as a proportion of at centre | [Ca^2+^]^TSR^_edge_ /[Ca^2+^]^TSR^_centre_ |  | 0.1407 | 0.1836 | 0.2629 | 0.3698 | 0.4671 |
| [Ca^2+^] at the SR Membrane | | | | | | | |
| At the centre of the T-SR junction | [Ca^2+^]^SR^_centre_ | μmol/dm^3^ | 22.3261 | 12.5739 | 5.6273 | 2.5548 | 1.4937 |
| At halfway between centre and edge of T-SR junction | [Ca^2+^]^SR^_50%_ | μmol/dm^3^ | 17.5995 | 10.074 | 4.6506 | 2.2017 | 1.3329 |
| At the edge of the T-SR junction | [Ca^2+^]^SR^_edge_ | μmol/dm^3^ | 3.3598 | 2.511 | 1.6538 | 1.0773 | 0.7979 |
| Concentration halfway as a proportion of at centre | [Ca^2+^]^SR^_50%_ / [Ca^2+^]^SR^_centre_ |  | 0.7883 | 0.8012 | 0.8264 | 0.8618 | 0.8924 |
| Concentration at edge as a proportion of at centre | [Ca^2+^]^SR^_edge_ / [Ca^2+^]^SR^_centre_ |  | 0.1505 | 0.1997 | 0.2939 | 0.4217 | 0.5342 |
| Relative SR/T membrane [Ca^2+^] | | | | | | | |
| At the centre of the T-SR junction | [Ca^2+^]^SR^_centre_ /[Ca^2+^]^T^_centre_ |  | 1.0206 | 1.0371 | 1.0869 | 1.2136 | 1.4264 |
| At halfway between centre and edge of T-SR junction | [Ca^2+^]^SR^_50%_/ [Ca^2+^]^T^_50%_ |  | 1.0262 | 1.0468 | 1.1071 | 1.2546 | 1.4887 |
| At the edge of the T-SR junction | [Ca^2+^]^SR^_edge_ /[Ca^2+^]^T^_edge_ |  | 1.1107 | 1.1528 | 1.2492 | 1.4320 | 1.6885 |
| Concentration differences between centre and edge | | | | | | |  |
| At the T-Tubular Membrane | [Ca^2+^]^ΔT^ | μmol/dm^3^ | 18.8513 | 9.9458 | 3.8535 | 1.3527 | 0.5746 |
| At halfway between the two membranes | [Ca^2+^]^ΔTSR^ | μmol/dm^3^ | 18.8954 | 9.9898 | 3.8989 | 1.3974 | 0.6170 |
| At the SR Membrane | [Ca^2+^]^ΔSR^ | μmol/dm^3^ | 18.9664 | 10.063 | 3.9735 | 1.4774 | 0.6958 |
| Radial distances for [Ca^2+^] to fall to 50% of centre value | | | | | | |  |
| At the T-Tubular membrane | *X_T_* | nm | 83.6667 | 62.2900 | 40.7413 | 26.2208 | 18.9997 |
| At halfway between the two membranes | *X_TSR_* | nm | 83.882 | 62.5751 | 41.141 | 26.6588 | 19.3418 |
| At the SR Membrane | *X_SR_* | nm | 84.5222 | 63.4282 | 42.3711 | 28.0986 | N/A |
| Radial distances to fall to 50% as a fraction of total TSR Radius | | | | | | | |
| At the T-Tubular membrane | 2*X*_T_ /*d* |  | 0.7606 | 0.7786 | 0.8148 | 0.8740 | 0.9500 |
| At halfway between the two membranes, | 2*X*_TSR_ /*d* |  | 0.7626 | 0.7822 | 0.8228 | 0.8886 | 0.9671 |
| At the SR Membrane, | 2*X*_SR_ /*d* |  | 0.7684 | 0.7929 | 0.8474 | 0.9366 | 1 |
| Radial distances for [Ca^2+^] to fall to 50% between centre and edge value | | | | | | |  |
| At the T-Tubular membrane | *X’_T_* | nm | 77.6670 | 56.4151 | 35.1250 | 20.8964 | 13.8396 |
| At halfway between the two membranes | *X’_TSR_* | nm | 77.7578 | 56.5389 | 35.3178 | 21.1447 | 14.0615 |
| At the SR Membrane | *X’_SR_* | nm | 77.9044 | 56.7452 | 35.6408 | 21.6303 | 14.5728 |

| **Supplementary Table S7: T-SR junction Ca^2+^ microdomains in resting muscle fibres** | | | | | | | |
| --- | --- | --- | --- | --- | --- | --- | --- |
| **State of Variables:** *Values held constant:* Boundary conditions: in vivo Ca^2+^ diffusion coefficient, *D* = 4 × 10^-7^ nm^2^/s; exit length, λ = 9.2 nm, determined as described in Table 2. Anatomical parameters as defined in Table 1. *Computational conditions:* Mesh size = 6 nm; stepsize = 500 ns; time resolution (number of steps/computational run) = 1000; computational endpoint = 500 μs. *Values varied*: flux density *J*_influx_ and corresponding Ca^2+^ influx, *Φ*_influx_ determined from [Ca^2+^]_edge._ | | | | | | | |
| **Name of Variable (Column)** | **Definition** | **Dimensions (Physiological)** | **Calcium flux densities into the T-SR space explored** | | | | |
| Resting cytosolic [Ca^2+^] explored | | | | | | | |
| Resting cytosolic [Ca2+] | [Ca^2+^] | μmol/dm^3^ | **0.038** | **0.300** | **0.060** | **0.140** |  |
| Boundary conditions over a range of varied test voltages: T-SR Junction | | | | | | | |
| Ca^2+^ flux density into T-SR junction | *J_i_*_nflux_ | mol/(nm^2^s) | 3.68 × 10^-26^ | 2.91 × 10^-25^ | 5.82 × 10^-26^ | 1.358 × 10^-25^ | |
| Ca^2+^ flux into T-SR junction | *Φ*_influx_ | mol/s | 1.3989 × 10^-21^ | 1.1062 × 10^-20^ | 2.2124 × 10^-21^ | 5.1622 × 10^-21^ | |
| Steady-state solutions, concentrations computed over time | | | | | | | |
| At the centre of the T-SR junction |  | μmol/dm^3^ | 0.2698 | 2.1334 | 0.4267 | 0.9956 | |
| At halfway between centre and edge of T-SR junction |  | μmol/dm^3^ | 0.2122 | 1.6778 | 0.3356 | 0.783 | |
| At the edge of the T-SR junction |  | μmol/dm^3^ | 0.0379 | 0.3 | 0.06 | 0.14 | |
| Computed steady-state concentration profiles | | | | | | | |
| [Ca^2+^] at the T-Tubular Membrane | | | | | | | |
| At the centre of the T-SR junction | [Ca^2+^]^T^_centre_ | μmol/dm^3^ | 0.2683 | 2.1213 | 0.4243 | 0.99 | |
| At halfway between centre and edge of T-SR junction | [Ca^2+^]^T^_50%_ | μmol/dm^3^ | 0.2103 | 1.663 | 0.3326 | 0.7761 | |
| At the edge of the T-SR junction | [Ca^2+^]^T^_edge_ | μmol/dm^3^ | 0.0371 | 0.2933 | 0.0587 | 0.1369 | |
| Concentration halfway as a proportion of at centre | [Ca^2+^]^T^_50%_ / [Ca^2+^]^T^_centre_ |  | 0.784 | 0.784 | 0.784 | 0.784 | |
| Concentration at edge as a proportion of at centre | [Ca^2+^]^T^_edge_ / [Ca^2+^]^T^_centre_ |  | 0.1383 | 0.1383 | 0.1383 | 0.1383 | |
| [Ca^2+^] halfway between the two membranes | | | | | | | |
| At the centre of the T-SR junction | [Ca^2+^]^TSR^_centre_ | μmol/dm^3^ | 0.2696 | 2.1322 | 0.4264 | 0.995 | |
| At halfway between centre and edge of T-SR junction | [Ca^2+^]^TSR^_50%_ | μmol/dm^3^ | 0.2117 | 1.674 | 0.3348 | 0.7812 | |
| At the edge of the T-SR junction | [Ca^2+^]^TSR^_edge_ | μmol/dm^3^ | 0.0379 | 0.3 | 0.06 | 0.14 | |
| Concentration halfway as a proportion of at centre | [Ca^2+^]^TSR^_50%_ / [Ca^2+^]^TSR^_centre_ |  | 0.7851 | 0.7851 | 0.7851 | 0.7851 | |
| Concentration at edge as a proportion of at centre | [Ca^2+^]^TSR^_edge_ / [Ca^2+^]^TSR^_centre_ |  | 0.1407 | 0.1407 | 0.1407 | 0.1407 | |
| [Ca^2+^] at the SR Membrane | | | | | | | |
| At the centre of the T-SR junction | [Ca^2+^]^SR^_centre_ | μmol/dm^3^ | 0.2738 | 2.165 | 0.433 | 1.0103 | |
| At halfway between centre and edge of T-SR junction | [Ca^2+^]^SR^_50%_ | μmol/dm^3^ | 0.2158 | 1.7067 | 0.3413 | 0.7965 | |
| At the edge of the T-SR junction | [Ca^2+^]^SR^_edge_ | μmol/dm^3^ | 0.0412 | 0.3258 | 0.0652 | 0.1521 | |
| Concentration halfway as a proportion of at centre | [Ca^2+^]^SR^_50%_ / [Ca^2+^]^SR^_centre_ |  | 0.7883 | 0.7883 | 0.7883 | 0.7883 | |
| Concentration at edge as a proportion of at centre | [Ca^2+^]^SR^_edge_ / [Ca^2+^]^SR^_centre_ |  | 0.1505 | 0.1505 | 0.1505 | 0.1505 | |
| Relative SR/T membrane [Ca^2+^] | | | | | | | |
| At the centre of the T-SR junction | [Ca^2+^]^SR^_centre_ /[Ca^2+^]^T^_centre_ |  | 1.0206 | 1.0206 | 1.0206 | 1.0206 | |
| At halfway between centre and edge of T-SR junction | [Ca^2+^]^SR^_50%_/ [Ca^2+^]^T^_50%_ |  | 1.0262 | 1.0262 | 1.0262 | 1.0262 | |
| At the edge of the T-SR junction | [Ca^2+^]^SR^_edge_ /[Ca^2+^]^T^_edge_ |  | 1.1108 | 1.1108 | 1.1108 | 1.1108 | |
| Concentration differences between centre and edge | | | | | | | |
| At the T-Tubular Membrane | [Ca^2+^]^ΔT^ | μmol/dm^3^ | 0.2312 | 1.828 | 0.3656 | 0.8531 | |
| At halfway between the two membranes | [Ca^2+^]^ΔTSR^ | μmol/dm^3^ | 0.2317 | 1.8323 | 0.3665 | 0.8551 | |
| At the SR Membrane | [Ca^2+^]^ΔSR^ | μmol/dm^3^ | 0.2326 | 1.8392 | 0.3678 | 0.8583 | |
| Radial distances for [Ca^2+^] to fall to 50% of centre value | | | | | | | |
| At the T-Tubular membrane | *X_T_* | nm | 83.6691 | 83.6691 | 83.6691 | 83.6691 | |
| At halfway between the two membranes | *X_TSR_* | nm | 83.8845 | 83.8845 | 83.8845 | 83.8845 | |
| At the SR Membrane | *X_SR_* | nm | 84.5249 | 84.5249 | 84.5249 | 84.5249 | |
| Radial distances for [Ca^2+^] to fall to 50% between centre and edge value | | | | | | | |
| At the T-Tubular membrane | *X’_T_* | nm | 77.6692 | 77.6692 | 77.6692 | 77.6692 | |
| At halfway between the two membranes | *X’_TSR_* | nm | 77.7601 | 77.7601 | 77.7601 | 77.7601 | |
| At the SR Membrane | *X’_SR_* | nm | 77.9066 | 77.9066 | 77.9066 | 77.9066 | |

Previous determinations of resting [Ca^2+^] (μM) in frog skeletal muscle; measurements using: Fura-2 conjugated to high molecular weight Dextran: 0.06 – 0.14 (ref. ^16^); ion selective electrodes/Aequorin (indirect method): 0.038 – 0.059 (ref. ^17^); Fura red: 0.18 – 0.27 (ref. ^18^); Fluo-3: 0.1 – 0.3 (ref. ^19^).

***Appendix for MATLAB Code***

**Parameter Specification**

**1) General Parameters**

% Clear the Workspace just to ensure all previous stored parameters have

% been removed and that my memory is clear before modelling

clear

clear global R

tic

% Get the current date and time (for file storage)

CurrentDateAndTime = datestr(now,'yyyy-mm-dd HH-MM');

% Radius of the Terminal SR (nm)

TSR_Radius = 110;

% Width of the T-SR Junction (nm)

TSR_Width = 12;

% Maximum Mesh Tetrahedral Length (nm)

Mesh_Hmax = 3;

% Flux Density across the T-Tubular Membrane (mol/nm2/s)

F1_Flux = 0;

% Flux Density across the Sarcoplasmic Reticulum Membrane (mol/nm2/s)

F2_Flux = 3e-24;

% Diffusion Coefficient (nm2/s)

D = 4e7;

% Modelling End Time (s)

T_End = 5e-4;

% Number of Time Points to Sample and Time Point Spacing

T_Resolution = 1000;

T_Spacing = T_End / T_Resolution;

**2) Edge Boundary Condition Options**

% Exit Length (nm)

Exit_Length = 9.2;

% Calculated co-efficient 'R' for Calcium Flux Density, passed onto loop at

% the end of code

global R

R = D/Exit_Length;

**Model Set-Up**

**1) Specifying Geometry**

% Produce a cylindrical geometry with dimensions specified above

gm = multicylinder(TSR_Radius,TSR_Width);

% Display geometry as assigned to PDE Model

model = createpde;

model.Geometry = gm;

% Figure display options

figure1 = figure;

pdegplot(model,'FaceAlpha',0.5,'FaceLabels',"off");

hold on

figure1.CurrentAxes.LineWidth = 2;

figure1.CurrentAxes.GridAlpha = 0.3;

figure1.CurrentAxes.FontSize = 12;

figure1.CurrentAxes.FontWeight = "bold";

xlabel('Distance (nm)','FontSize',12);

ylabel('Distance (nm)','FontSize',12);

zlabel('Distance (nm)','FontSize',12);

title('Geometry of the T-SR Junction')

figure1.CurrentAxes.Title.FontSize = 12;

zlim([-60,60])

xlim([-TSR_Radius,TSR_Radius])

ylim([-TSR_Radius,TSR_Radius])

grid on

hold off

**2) Meshing**

% Generate a mesh on the geometry with properties described above

meshgeom = generateMesh(model,"Hmax",Mesh_Hmax);

% Copy a component of matrix meshgeom to noderet for figure production

% later

noderet=meshgeom.Nodes;

% Figure display of meshed geometry

figure2 = figure;

pdeplot3D(model,"ElementLabels","off");

hold on

figure2.CurrentAxes.FontSize = 12;

title('Finite element mesh for analysis',"FontSize",12)

hold off

**3) Boundary Conditions**

% Apply a 0 flux across the T-tubular membrane

applyBoundaryCondition(model,"neumann","Face",1,"g",F1_Flux,"q",0);

% Apply a flux across the SR membrane equal to that specified in initial

% conditions

applyBoundaryCondition(model,"neumann","Face",2,"g",F2_Flux,"q",0);

% Apply a flux across the edge membrane equal to the output of function

% below

applyBoundaryCondition(model,"neumann","Face",3,"g",@F3_flux,"q",0,"Vectorized","off");

**4) Initial Conditions**

% Set initial PDE conditions to 0

setInitialConditions(model,0,0);

**5) Partial Differential Equation Coefficients**

%Tell system this is a parabolic by setting m=0, d=1

specifyCoefficients(model,"c",D,"f",0,"a",0,"d",1,"m",0);

**Solution**

**1) Time for Solution**

%tlist is the initial time : time spacing : end time in seconds

tlist = 0:T_Spacing:T_End;

**2) Generating Solution**

solution = solvepde(model,tlist);

**3) Solution Conversion**

%Convert Nodal solution into uM from mol/nm3, saved as sol

sol = solution.NodalSolution*1e30;

timeForSolution = toc

**4) Saving the Solution to File**

FileName = sprintf('results_%s.mat', CurrentDateAndTime);

FileName2 = sprintf('results2_%s.mat', CurrentDateAndTime);

FolderName = sprintf(CurrentDateAndTime);

TotalFileName = fullfile('E:\','Documents','MATLAB','Examples','R2020b','pde','resultsfolder',FolderName,FileName);

TotalFileName2 = fullfile('E:\','Documents','MATLAB','Examples','R2020b','pde','resultsfolder',FolderName,FileName2);

mkdir(fullfile('E:\','Documents','MATLAB','Examples','R2020b','pde','resultsfolder',FolderName));

mkdir(fullfile('E:\','Documents','MATLAB','Examples','R2020b','pde','resultsfolder',FolderName,'Previews'));

save(TotalFileName,'*',"-v7.3")

**Data Presentation**

**1) General Heat Map**

% Display a general heat map for the solution using PDE Toolbox

figure3 = figure;

pdeplot3D(model,"ColorMapData",sol(:,end),"Mesh","on");

hold on

figure3.CurrentAxes.FontSize = 12;

title({'Heat map: radial [Ca^{2+}] distribution,','T-tubular membrane face'})

heatbar1 = colorbar;

heatbar1.Label.String = 'Concentration (\muM)';

heatbar1.FontSize = 12;

heatbar1.Label.FontSize = 12;

hold off

% Display this again but with different orientation

figure3_1 = figure;

pdeplot3D(model,"ColorMapData",sol(:,end),"Mesh","on");

hold on

figure3_1.CurrentAxes.FontSize = 12;

view(142.5,-30)

title({'Heat map: radial [Ca^{2+}] distribution,','sarcoplasmic reticular membrane face'})

heatbar1_1 = colorbar;

heatbar1_1.Label.String = 'Concentration (\muM)';

heatbar1_1.FontSize = 12;

heatbar1_1.Label.FontSize = 12;

hold off

**2) Heat Map top and bottom views**

figure4 = figure;

pdeplot3D(model,'ColorMapData',sol(:,end),'Mesh',"on");

hold on

figure4.CurrentAxes.FontSize = 12;

title({'Heat map: radial [Ca^{2+}] distribution,','sarcoplasmic reticular membrane face'})

view(2)

heatbar2 = colorbar;

heatbar2.Label.String = 'Concentration (\muM)';

heatbar2.FontSize = 11;

heatbar2.Label.FontSize = 11;

hold off

figure5 = figure;

pdeplot3D(model,'ColorMapData',sol(:,end),'Mesh',"on");

hold on

figure5.CurrentAxes.FontSize = 12;

title({'Heat map: radial [Ca^{2+}] distribution,','T-tubular membrane face'})

view(0,-90)

heatbar3 = colorbar;

heatbar3.Label.String = 'Concentration (\muM)';

heatbar3.FontSize = 11;

heatbar3.Label.FontSize = 11;

hold off

**3) Concentration vs time for a node**

Obtain and plot value of Concentration at a specific point

% Co-ordinates of points to sample [x,y,z]

nidcoords1 = [TSR_Radius,0,0.5*TSR_Width];

nidcoords2 = [0.5*TSR_Radius,0,0.5*TSR_Width];

nidcoords3 = [0,0,0.5*TSR_Width];

% 3D Obtain Node function

getClosestNode = @(p,x,y,z) min((p(1,:) - x).^2 + (p(2,:) - y).^2 + (p(3,:) - z).^2);

% Assigns the closest node to the data points x,y,z to variable 'nid'

[~,nid1]=getClosestNode(meshgeom.Nodes,nidcoords1(1),nidcoords1(2),nidcoords1(3));

[~,nid2]=getClosestNode(meshgeom.Nodes,nidcoords2(1),nidcoords2(2),nidcoords2(3));

[~,nid3]=getClosestNode(meshgeom.Nodes,nidcoords3(1),nidcoords3(2),nidcoords3(3));

% Find concentration at that point at different times and plot it

figure6 = figure;

plot(tlist,sol(nid1,:),'k',"LineWidth",2);

hold on

plot(tlist,sol(nid2,:),'r',"LineWidth",2)

plot(tlist,sol(nid3,:),'b',"LineWidth",2)

figure6.CurrentAxes.LineWidth = 2;

figure6.CurrentAxes.FontSize = 12;

grid off

legend('At the edge','Half-way between the centre and edge of T-SR space','At centre of T-SR space',"Location","southoutside")

title('[Ca^{2+}]: time dependences',' ')

xlabel('Time (s)')

ylabel('Concentration (\muM)')

xlim([0 T_End])

%ylim([0 25])

box off

hold off

% Display and save the concentrations at the end time, considered steady

% state

Css_edge = interp1(tlist,sol(nid1,:),T_End);

Css_mid = interp1(tlist,sol(nid2,:),T_End);

Css_centre = interp1(tlist,sol(nid3,:),T_End);

**4) Concentration vs radial distance**

% Create a grid of Values

xradial = 0:1:TSR_Radius;

yradial = 0*ones(1,length(xradial));

zradial = yradial;

% Interpolate the solution across this solution grid

interpolate_radialplot = interpolateSolution(solution,xradial,yradial,zradial,1:length(tlist))*1e30;

% Produce figures with this new interpolation

figure7 = figure;

plot(xradial,interpolate_radialplot(:,end),'Color','k',"LineWidth",2);

hold on

figure7.CurrentAxes.LineWidth = 2;

figure7.CurrentAxes.FontSize = 12;

figure7.CurrentAxes.XTick = [0:10:TSR_Radius];

grid off

title('Steady state [Ca^{2+}]: position dependences')

xlabel('Distance from the centre of the geometry (nm)')

ylabel('Concentration (\muM)')

xlim([0,TSR_Radius])

%ylim([0,25])

box off

hold off

zradial2 = 0.5*TSR_Width*ones(1,length(xradial));

zradial3 = TSR_Width*ones(1,length(xradial));

interpolate_radialplot2 = interpolateSolution(solution,xradial,yradial,zradial2,1:length(tlist))*1e30;

interpolate_radialplot3 = interpolateSolution(solution,xradial,yradial,zradial3,1:length(tlist))*1e30;

figure7_1 = figure;

plot(xradial,interpolate_radialplot(:,end),'Color','k',"LineWidth",2);

hold on

plot(xradial,interpolate_radialplot2(:,end),'Color','r',"LineWidth",2);

plot(xradial,interpolate_radialplot3(:,end),'Color','b',"LineWidth",2);

figure7_1.CurrentAxes.FontSize = 12;

figure7_1.CurrentAxes.LineWidth = 2;

figure7_1.CurrentAxes.XTick = [0:10:TSR_Radius];

grid off

xlim([0,TSR_Radius])

%ylim([0,25])

title('Steady state [Ca^{2+}]: position dependences',' ')

xlabel('Distance from the centre of the geometry (nm)')

ylabel('Concentration (\muM)')

box off

legend('At the T-Tubular Membrane','Half-way between the SR and T-Tubular Membrane','At the Sarcoplasmic Reticulum Membrane',"Location","southoutside")

hold off

CentreCa_Tmem = interp1(xradial,interpolate_radialplot(:,end),0);

CentreCa_Half = interp1(xradial,interpolate_radialplot2(:,end),0);

CentreCa_SRmem = interp1(xradial,interpolate_radialplot3(:,end),0);

MiddleCa_Tmem = interp1(xradial,interpolate_radialplot(:,end),0.5*TSR_Radius)

MiddleCa_Half = interp1(xradial,interpolate_radialplot2(:,end),0.5*TSR_Radius)

MiddleCa_SRmem = interp1(xradial,interpolate_radialplot3(:,end),0.5*TSR_Radius)

EdgeCa_Tmem = interp1(xradial,interpolate_radialplot(:,end),TSR_Radius);

EdgeCa_Half = interp1(xradial,interpolate_radialplot2(:,end),TSR_Radius);

EdgeCa_SRmem = interp1(xradial,interpolate_radialplot3(:,end),TSR_Radius);

RangeCa_Tmem = CentreCa_Tmem - EdgeCa_Tmem;

RangeCa_Half = CentreCa_Half - EdgeCa_Half;

RangeCa_SRmem = CentreCa_SRmem - EdgeCa_SRmem;

% Calculating Distance at which the concentration has fallen to half of its

% maximum value

DistHalfCa_Tmem = interp1(interpolate_radialplot(:,end),xradial,0.5*CentreCa_Tmem);

DistHalfCa_Half = interp1(interpolate_radialplot2(:,end),xradial,0.5*CentreCa_Half);

DistHalfCa_SRmem = interp1(interpolate_radialplot3(:,end),xradial,0.5*CentreCa_SRmem);

% Calculating Distance at which the concentration has fallen to half of its

% range

DistHalfRangeCa_Tmem = interp1(interpolate_radialplot(:,end),xradial,(0.5*RangeCa_Tmem+EdgeCa_Tmem));

DistHalfRangeCa_Half = interp1(interpolate_radialplot2(:,end),xradial,(0.5*RangeCa_Half+EdgeCa_Half));

DistHalfRangeCa_SRmem = interp1(interpolate_radialplot3(:,end),xradial,(0.5*RangeCa_SRmem+EdgeCa_SRmem));

**5) 3D Plot of Concentration at the T-Tubular Membrane**

% Specify time in modelling to generate the mesh

T_For_3D_Plot = T_Resolution + 1;

% Create a grid of values

[xmesh,ymesh,zmesh]=meshgrid(-TSR_Radius:TSR_Radius,-TSR_Radius:TSR_Radius,0);

interpolate_meshTtub = interpolateSolution(solution,xmesh,ymesh,zmesh,T_For_3D_Plot)*1e30;

interpolate_meshTtub = reshape(interpolate_meshTtub,size(xmesh));

% Surf plot

figure8 = figure;

surf(-TSR_Radius:TSR_Radius,-TSR_Radius:TSR_Radius,interpolate_meshTtub,'EdgeColor',"none");

hold on

figure8.CurrentAxes.LineWidth = 2;

figure8.CurrentAxes.GridAlpha = 0.3;

figure8.CurrentAxes.FontSize = 12;

figure8.CurrentAxes.YTick = [-100:50:100];

figure8.CurrentAxes.XTick = [-100:50:100];

figure8.CurrentAxes.ZTick = [0:5:25];

surfbar1 = colorbar;

surfbar1.Label.String = 'Concentration (\muM)';

surfbar1.FontSize = 12;

surfbar1.Label.FontSize = 12;

colormap("jet")

title({'Steady state [Ca^{2+}] map:','T-tubular membrane face'},' ')

xlabel('Position along X-axis of Geometry (nm)')

ylabel('Position along Y-axis of Geometry (nm)')

zlabel('Concentration (\muM)')

xlim([-TSR_Radius,TSR_Radius])

ylim([-TSR_Radius,TSR_Radius])

hold off

figure9 = figure;

surf(-TSR_Radius:TSR_Radius,-TSR_Radius:TSR_Radius,interpolate_meshTtub,'EdgeColor',"none");

hold on

figure9.CurrentAxes.LineWidth = 2;

figure9.CurrentAxes.GridAlpha = 0.3;

figure9.CurrentAxes.FontSize = 12;

surfbar2 = colorbar;

surfbar2.Label.String = 'Concentration (\muM)';

surfbar2.FontSize = 12;

surfbar2.Label.FontSize = 12;

view(90,0)

grid off

colormap("jet")

title({'Steady state [Ca^{2+}] along','T-tubular membrane face'},' ')

xlabel('Position along X-axis of Geometry (nm)')

ylabel('Position along Y-axis of Geometry (nm)')

zlabel('Concentration (\muM)')

ylim([-120,120])

%yticks([-100,0,100])

zlim([0 25])

hold off

**6) 2D Radial Colormap**

Slice_Plot_Resolution = 0.25

T_For_Slice_Plot = T_Resolution + 1;

[xslice,zslice]=meshgrid(-TSR_Radius:Slice_Plot_Resolution:TSR_Radius,linspace(0,TSR_Width,((1/Slice_Plot_Resolution)*2*TSR_Radius+1)));

yslice = 0*ones([length(xslice)]);

interpolate_radialplot = interpolateSolution(solution,xslice,yslice,zslice,T_For_Slice_Plot)*1e30;

interpolate_radialplot = reshape(interpolate_radialplot,size(xslice));

figure10 = figure;

imagesc(interpolate_radialplot,"XData",xslice(1,:),'YData',zslice(:,1));

hold on

figure10.CurrentAxes.FontSize = 12;

colormap("jet");

title({'Steady state [Ca^{2+}]','through midline slice'})

xlabel('X displacement (nm)')

ylabel('Distance from T-Tubular Membrane (nm)')

yticks = [-110:55:110];

slicebar=colorbar;

slicebar.FontSize = 12;

slicebar.Label.String='Concentration (\muM)';

slicebar.Label.FontSize = 12;

hold off

**Calculate Calcium Flux**

Entry Molar Flux = Molar Flux Density * F2 Area

Entry_MF = F2_Flux*pi*TSR_Radius^2;

Exit Molar Flux = Molar Flux Density * F3 Area = R*C*F3 Area

global R

Exit_MF = R*Css_edge*10^-30*pi*2*TSR_Radius*TSR_Width;

**Function for F3 Flux**

function f = F3_flux(~,state) % Specify function F3_Flux retrieves the solution state at that time

global R

if isnan(state.u) % Save computation time and errors through setting the flux equal to NaN where the solution is NaN

f = NaN;

else

f = -R*state.u; % At all values of concentration at particular called position the value of flux density from that surface is R*u

end

end

***List of abbreviations***

*a*, fibre diameter;

*A*_S_, sarcomeric surface membrane area;

*A*_T_, sarcomeric tubular membrane surface area;

*A*_TSR_, total sarcomeric T-tubular membrane area contributing to T-SR junctions;

*b*, spatially dependent coefficient: MATLAB solution for PDE;

BC, boundary condition;

*c*, diffusional term: MATLAB solution for PDE;

$\left[ Ca^{2+} \right],$ Ca^2+^ concentration;

$\left[ Ca^{2+} \right]_{\mathrm{edge}}$, $\left[ Ca^{2+} \right] at edge of T-SR junction;$

$$\left[ Ca^{2+} \right]_{\mathrm{TSR}},\left[ Ca^{2+} \right] within T-SR junction;$$

[Ca^2+^]_max_ , experimental peak cytosolic Ca^2+^ concentration;

[Ca^2+^]^TSR^_edge,_ Ca^2+^ concentration at the edge of the T-SR junction;

Ca^2+^/CaM, Ca^2+^-calmodulin;

CaM, calmodulin;

CaMKII, Ca^2+^/CaM-regulated CaM kinase II;

Cav1.1, skeletal muscle Ca^2+^ channel;

Cav1.2, cardiac muscle Ca^2+^ channel;

centre (subscript: denoting radial position in T-SR junction), at centre of T-SR junction;

CPU, central processor unit;

8-CPT, 8‐(4‐chlorophenylthio)‐2′‐O‐methyladenosine‐3′,5′‐cyclic monophosphate;

*C*_S_, surface membrane capacitance normalized to unit area of surface membrane;

*C*_T_, tubular membrane capacitance normalized to unit area of surface membrane;

*D,* diffusion coefficient;

*d*, radial diameter of SR membrane in a single T-SR junction;

3D, three-dimensional;

d[Ca^2+^]/d*t*, experimental rate of SR Ca^2+^ release;

DHPR, dihydropyridine receptor;

DHPR1, skeletal muscle dihydropyridine receptor;

DHPR2, cardiac muscle dihydropyridine receptor;

ΔSR (superscript), differences between centre and edge at the SR membrane;

ΔT (superscript), differences between centre and edge at the T-membrane;

ΔTSR (superscript), differences between centre and edge at the midpoint between T- and SR membrane;

*E*, membrane potential;

edge (subscript: denoting radial position in T-SR junction), at edge of T-SR junction;

*f,* spatially dependent coefficient: MATLAB solution for PDE;

FEM, finite element method;

50% (subscript: denoting radial position in T-SR junction), equidistant between the centre and edge of the T-SR junction;

*g*, spatially dependent coefficient: MATLAB solution for PDE;

*h*, spatially dependent coefficient: boundary flux term for the Neumann condition in MATLAB solution for PDE;

HEK293, human embryonic kidney cell line 293

*I*_Na_, Na^+^ current;

IQ, isoleucine-glutamine domain;

$J_{\mathrm{efflux}}$, Ca^2+^ efflux density from a single T-SR junction;

*J*_influx_, Ca^2+^ influx density into a single T-SR junction;

*l*, sarcomere length;

*λ*, exit length;

*m*, spatially dependent coefficient: MATLAB solution for PDE;

Nav, Na^+^ channel;

Na_v_1.4, skeletal muscle Na^+^ channel;

Na_v_1.5, cardiac muscle Na^+^ channel;

*N*_TSR,_ number of T-SR junctions in unit muscle volume;

*ξ,* proportion of total tubular membrane area *A*_T_ accounted for by T-SR junctions;

ODE, ordinary differential equation;

PDE, partial differential equation;

RAM, random access memory;

RyR, ryanodine receptor;

RyR1, skeletal muscle ryanodine receptor;

RyR2, cardiac muscle ryanodine receptor;

S, surface membrane;

SERCA, SR Ca^2+^-ATPase;

SR (superscript: denoting axial position in T-SR junction), at the SR membrane within the T-SR junction;

SR, sarcoplasmic reticulum/reticular;

T (superscript: denoting axial position in T-SR junction), at the T-tubular membrane within the T-SR junction;

T-, transverse;

T, tubular membrane;

TSR (superscript: denoting axial position in T-SR junction), at halfway along axial T-SR distance;

*u*, spatially dependent solution; MATLAB solution for PDE;

*Φ*_efflux_, Ca^2+^ efflux from a single T-SR junction;

$\Phi_{\mathrm{influx}}$, Ca^2+^ influx into a single T-SR junction;

*V,* sarcomere cytosolic volume;

*w*, axial T-SR distance in a single T-SR junction;

*X*, radial T-SR distance for [Ca^2+^] to fall to 50% of centre value;

*X*’, radial T-SR distances for [Ca^2+^] to fall to 50% between centre and edge value;

*χ,* the maximum element length (FEM).

***References cited in supplementary file***

1. Kovacs, L., Rios, E. & Schneider, M. F. Measurement and modification of free calcium transients in frog skeletal muscle fibres by a metallochromic indicator dye. *J. Physiol.* **343,** 161–196 (1983).

2. Vanýsek, P. Ionic conductivity and diffusion at infinite dilution. In: Handbook of chemistry and physics,. *CRC Handb. Chem. Physics, Ed. 91* **96,** 5–98 (2002).

3. Donahue, B. S. & Abercrombie, R. F. Free diffusion coefficient of ionic calcium in cytoplasm. *Cell Calcium* **8,** 437–448 (1987).

4. Baylor, S. M. & Hollingworth, S. Model of sarcomeric Ca2+ movements, including ATP Ca2+ binding and diffusion, during activation of frog skeletal muscle. *J. Gen. Physiol.* **112,** 297–316 (1998).

5. Baylor, S. M., Hollingworth, S. & Chandler, W. K. Comparison of simulated and measured calcium sparks in intact skeletal muscle fibers of the frog. *J. Gen. Physiol.* **120,** 349–368 (2002).

6. Kushmerick, M. J. & Podolsky, R. J. Ionic mobility in muscle cells. *Science (80-. ).* **166,** 1297–1298 (1969).

7. Wang, J. H. Tracer-diffusion in Liquids. IV. Self-diffusion of Calcium Ion and Chloride Ion in Aqueous Calcium Chloride Solutions. *Journal of the American Chemical Society* **75,** 1769–1770 (1953).

8. Nasi, E. & Tillotson, D. The rate of diffusion of Ca2+ and Ba2+ in a nerve cell body. *Biophys. J.* **47,** 735–738 (1985).

9. Al-Baldawi, N. F. & Abercrombie, R. F. Calcium diffusion coefficient in Myxicola axoplasm. *Cell Calcium* **17,** 422–430 (1995).

10. Cordeiro, J. M. *et al.* Location of the initiation site of calcium transients and sparks in rabbit heart Purkinje cells. *J. Physiol.* **531,** 301–314 (2001).

11. Swietach, P., Spitzer, K. W. & Vaughan-Jones, R. D. Modeling calcium waves in cardiac myocytes: Importance of calcium diffusion. *Front. Biosci.* **15,** 661–680 (2010).

12. Franzini-Armstrong, C. Studies of the triad: I. Structure of the junction in frog twitch fibers. *J. Cell Biol.* **47,** 488–499 (1970).

13. Martin, C. A. *et al.* The effect of extracellular tonicity on the anatomy of triad complexes in amphibian skeletal muscle. *J. Muscle Res. Cell Motil.* **24,** 407–415 (2003).

14. Chawla, S., Skepper, J. N., Hockaday, A. R. & Huang, C. L.-H. Calcium waves induced by hypertonic solutions in intact frog skeletal muscle fibres. *J. Physiol.* **536,** 351–9 (2001).

15. Usher-Smith, J. A., Fraser, J. A., Huang, C. L.-H. & Skepper, J. N. Alterations in triad ultrastructure following repetitive stimulation and intracellular changes associated with exercise in amphibian skeletal muscle. *J. Muscle Res. Cell Motil.* **28,** 19–28 (2007).

16. Konishi, M. & Watanabe, M. Resting cytoplasmic free Ca2+ concentration in frog skeletal muscle measured with fura-2 conjugated to high molecular weight dextran. *J. Gen. Physiol.* **106,** 1123–1150 (1995).

17. Blatter, L. A. & Blinks, J. R. Simultaneous measurement of Ca2+ in muscle with Ca electrodes and aequorin: Diffusible cytoplasmic constituent reduces Ca2+-independent luminescence of aequorin. *J. Gen. Physiol.* **98,** 1141–1160 (1991).

18. Kurebayashi, N., Harkins, A. B. & Baylor, S. M. Use of fura red as an intracellular calcium indicator in frog skeletal muscle fibers. *Biophys. J.* **64,** 1934–1960 (1993).

19. Harkins, A. B., Kurebayashi, N. & Baylor, S. M. Resting myoplasmic free calcium in frog skeletal muscle fibers estimated with fluo-3. *Biophys. J.* **65,** 865–881 (1993).
